# Approach for Propagating Radiometric Data Uncertainties Through NASA Ocean Color Algorithms

**DOI:** 10.3389/feart.2019.00176

**Published:** 2019-07-18

**Authors:** Lachlan I. W. McKinna, Ivona Cetinić, Alison P. Chase, P. Jeremy Werdell

**Affiliations:** 1 Go2Q Pty Ltd., Buderim, QLD, Australia; 2 Ocean Ecology Laboratory, NASA Goddard Space Flight Center, Greenbelt, MD, United States; 3 GESTAR/Universities Space Research Association, Columbia, MD, United States; 4 School of Marine Sciences, University of Maine, Orono, ME, United States

**Keywords:** ocean color, remote sensing, bio-optics, uncertainties, oceanography, radiometry, biogeochemistry

## Abstract

Spectroradiometric satellite observations of the ocean are commonly referred to as “ocean color” remote sensing. NASA has continuously collected, processed, and distributed ocean color datasets since the launch of the Sea-viewing Wide-field-of-view Sensor (SeaWiFS) in 1997. While numerous ocean color algorithms have been developed in the past two decades that derive geophysical data products from sensor-observed radiometry, few papers have clearly demonstrated how to estimate measurement uncertainty in derived data products. As the uptake of ocean color data products continues to grow with the launch of new and advanced sensors, it is critical that pixel-by-pixel data product uncertainties are estimated during routine data processing. Knowledge of uncertainties can be used when studying long-term climate records, or to assist in the development and performance appraisal of bio-optical algorithms. In this methods paper we provide a comprehensive overview of how to formulate first-order first-moment (FOFM) calculus for propagating radiometric uncertainties through a selection of bio-optical models. We demonstrate FOFM uncertainty formulations for the following NASA ocean color data products: chlorophyll-a pigment concentration (*Chl*), the diffuse attenuation coefficient at 490 nm (*K*_*d*,490_), particulate organic carbon (*POC*), normalized fluorescent line height (*nflh*), and inherent optical properties (IOPs). Using a quality-controlled *in situ* hyperspectral remote sensing reflectance (*R*_*rs*,*i*_) dataset, we show how computationally inexpensive, yet algebraically complex, FOFM calculations may be evaluated for correctness using the more computationally expensive Monte Carlo approach. We compare bio-optical product uncertainties derived using our test *R*_*rs*_ dataset assuming spectrally-flat, uncorrelated relative uncertainties of 1, 5, and 10%. We also consider spectrally dependent, uncorrelated relative uncertainties in *R*_*rs*_. The importance of considering spectral covariances in *R*_*rs*_, where practicable, in the FOFM methodology is highlighted with an example SeaWiFS image. We also present a brief case study of two *POC* algorithms to illustrate how FOFM formulations may be used to construct measurement uncertainty budgets for ecologically-relevant data products. Such knowledge, even if rudimentary, may provide useful information to end-users when selecting data products or when developing their own algorithms.

## INTRODUCTION

NASA has continually collected, processed, archived, and distributed global ocean color data since the launch of the Sea-viewing Wide Field-of-View Sensor (SeaWiFS) in 1997. This two decades-long multi-sensor data climatology continues to provide unprecedented synoptic-scale insight into near-surface oceanographic processes. Some of the satellite-derived variables, such as chlorophyll-a pigment concentration *Chl* (mg m^−3^), are considered as Essential Climate Variables (ECV) and are widely used by the oceanographic community to study phytoplankton ecology, marine biogeochemistry, and ecosystem responses to climate change (IOCCG, 2008;McClain, 2009; Franz et al., 2017).

Following formal definitions outlined in the Guide to Uncertainty in Measurement (JCGM, 2008), we can outline the objective of ocean color remote sensing as, to *measure* oceanographic quantities or *measurands*. We note that the *measurement procedure* involves a number of mathematical steps and assumptions that derive the *measurand* from sensor-observed top-of-atmosphere radiances. Thus, a derived ocean color data product is a *result of measurement* and should always be treated as an estimate of the *measurand* which has inherent *uncertainty*.

Quantifying uncertainty in derived ocean color data products (i.e.,measurands) is highly valuable, allowing end-users to: assess if datasets are fit-for-purpose, assess if observed temporal change is greater than uncertainty, assimilate uncertainties into climate models, and assess consistency among sensors (Maritorena et al., 2010; Gould et al., 2014). Additionally, a thorough understanding of uncertainty sources within a model may help guide the decisions of scientists when developing new satellite algorithms.

The measurement uncertainty (*u*_*measurement*_), in an ocean color data product, *y*, can be expressed as the following:

(1) $$u_{measurement}(y)=\sqrt{u_{data}^{2}(y)+u_{\text{mod}el}^{2}(y)},$$

where *u*_*model*_(*y*) represents uncertainties in *y* due to inherent inaccuracies/limitations in the algorithm (e.g., model coefficients), and *u*_*data*_(*y*) represents uncertainties in *y* due to uncertainties in sensor-observed radiometry (data). In this paper we focus on *u*_*data*_(*y*), that is, the propagation of radiometric uncertainties through bio-optical algorithms. For brevity, we shorten *u*_*data*_(*y*) to *u*(*y*) throughout this paper unless otherwise stated.

For the ocean color community, much of our understanding of measurement uncertainty in derived data products is sourced from validation exercises using in situ datasets (Bailey and Werdell, 2006; Antoine et al., 2008; Melin, 2010; Mélin et al., 2016) or from Monte Carlo-type simulations (Wang et al., 2005). We note that advanced statistical methodologies have also emerged for predicting uncertainties in derived ocean color products (Moore et al., 2009; Salama et al., 2009; Jay et al., 2018). While validation studies remain critical for appraising the absolute skill of an ocean color algorithm, such datasets themselves have their own measurement uncertainty associated with in situ observations (including uncertainties associated with subpixel temporal/spatial/environmental variability). Monte Carlo-type analyses are particularly useful for understanding measurement uncertainty, however, these approaches can be computationally expensive and are impracticable to implement within pixel-by-pixel ocean color processing.

More recently, analytical first-order first moment (FOFM) methods have been proposed that can directly propagate radiometric uncertainty through an ocean color algorithm to estimate derived data product uncertainty (Neukermans et al., 2009; Salama et al., 2009, 2011; Lee et al., 2010; Maritorena et al., 2010; Lamquin et al., 2013; Qi et al., 2017). These approaches are based on the *law of propagation of uncertainty* according to JCGM (2008). A FOFM methodology benefits from being computationally efficient, thereby allowing it to be implemented in pixel-by-pixel ocean color data processing software (Lamquin et al., 2013). In addition, FOFM calculations can be used to estimate the relative contribution of individual sources to total measurement uncertainty.

Work presented here is the first comprehensive examination of methods that can be used to estimate uncertainties in NASA’s standard bio-optical data products. In this study we aim to demonstrate the feasibility of using a FOFM uncertainty framework to approximate ocean color data uncertainty in derived data products. The FOFM method, which itself is an analytical approximation, is first appraised by comparing FOFM-derived uncertainties with Monte Carlo-derived uncertainties. We demonstrate how this approach can be used as a method to check the correctness of FOFM calculations. Second, using FOFM propagation theory, we estimate uncertainty in derived ocean color products given spectrally-flat, uncorrelated relative uncertainties of 1, 5, and 10% in spectral remote-sensing reflectances, *R*_*rs*,*i*_ (sr^−1^). We also consider spectrally-dependent, uncorrelated relative uncertainties in *R*_*rs*,*i*_ published by Hu et al. (2013). Third, we consider how inclusion of covariances affect uncertainty estimates. A sample SeaWiFS scene of the Hawaiian Islands is used in this case study. Finally, we demonstrate how the FOFM approach may be used to estimate measurement uncertainty budgets. In our case study we consider two algorithms for estimating particulate organic carbon (*POC*; mg m^−3^), a key metric used to understand oceanic biomass and the carbon cycle.

In this work, we utilize a high quality *in situ* hyperspectral *R*_*rs*,*i*_ dataset that can be spectrally subsampled to match the spectral characteristics of most existing and future ocean color sensors. This includes NASA’s Plankton, Aerosol, Cloud, ocean Ecosystem (PACE) mission that is currently under development and will carry the first dedicated hyperspectral ocean color sensor.

## DATA AND METHODS

### Bio-optical Algorithms and Data Products

The NASA Ocean Biology Data Archive and Active Distribution Center (OB.DAAC) distribute a number of derived marine data products in two separate data suites: (i) the standard ocean color (OC) data product suite and, (ii) the inherent optical properties (IOP) product suite. The OC suite comprises established (legacy) ocean color data products that were developed during the SeaWiFS (1997–2010) and Moderate Resolution Imaging Spectroradiometer aboard Aqua (MODISA 2002–present) missions. The IOP suite comprises spectral component absorption and backscattering coefficients derived using the default configuration of the Generalized Inherent Optical Properties (GIOP) algorithm framework (Werdell et al., 2013). A selection of the OC suite and IOP suite products were used in this study (Table 1). More comprehensive detail of the bio-optical algorithms used to derive these data products and their associated uncertainties are given in Appendices A–E (Supplementary Material). We note that in this study the GIOP used a spectral subset of our *R*_*rs*_ evaluation dataset (described in section Evaluation *R*_*rs*_ Dataset) spanning 412–655 nm.

### Modeling Bio-Optical Data Product Uncertainty

In this study we used the analytical law of propagation of uncertainty (JCGM, 2008) to propagate radiometric uncertainties through models used to derive bio-optical quantities. We follow the notation conventions outlined by JCGM (2008) where the uncertainty of a measured quantity, *y*, is denoted as *u*(*y*) and is the positive square root of the variance, *u*^2^(*y*). We note that *y* is derived from a model, *f*, of *N* input quantities, *x*_*i*_. Following (JCGM, 2008), for uncorrelated input quantities, *u*^2^(*y*) can be calculated as:

(2) $$u^{2}(y)=\sum_{i=1}^{N}{\left(\frac{\partial f}{\partial x_{i}}\right)}^{2}u^{2}\left(x_{i}\right)$$

where, *u*(*x*_*i*_) is the 1-σ uncertainty in the input quantity *x*_*i*_. For our notation of spectral properties used in ocean color remote sensing, subscripts *i* correspond to wavelength. In this study, partial derivatives of target parameters were calculated analytically, however, these could also be computed numerically. For the situation where uncertainties of input quantities are correlated, Equation 2 is extended to:

(3) $$u^{2}\left(y\right)=\sum_{i=1}^{N}{\left(\frac{\partial f}{\partial x_{i}}\right)}^{2}u^{2}\left(x_{i}\right)+2\sum_{i=1}^{N-1}\sum_{j=i+1}^{N}\frac{\partial f}{x_{i}}\frac{\partial f}{x_{j}}u\left(x_{i},x_{j}\right)$$

where u(*x*_*i*_, *x*_*j*_) = u(*x*_*j*_, *x*_*i*_) denotes the estimated error covariance associated with the quantities *x*_*i*_ and *x*_*j*_. Comprehensive details of partial derivative calculations for each bio-optical algorithm in Table 1 are given in Appendices A–E (Supplementary Material).

Monte Carlo (MC) methods are routinely used to perform sensitivity analyses as well as quantify model output uncertainties (Refsgaard et al., 2007). In this study, we have utilized a MC approach to appraise FOFM calculations. As the partial derivative calculus within FOFM uncertainty estimates can be complex, we have used MC-to-FOFM comparisons as a means of checking calculations.

The MC estimates of uncertainties in this study were computed as follows:

- A given bio-optical model, *f*, that derives an output *y*, that is considered a function of *n* spectral remote sensing reflectance bands, *R*_*rs*,*i*_, is run 5,000 times.
- Upon each iteration, each *R*_*rs*,*i*_ is perturbed by a factor Δ*r*_,*i*_ which is randomly sampled from a Gaussian distribution Δ*r*_,*i*_ ∼*N*(0,*u*(*R*_*rs*,*i*_)), in which the mean is zero and the standard deviation, u(*R*_*rs*,*i*_), is known or assumed. No spectral correlations are assumed.
- The MC simulation then generates a probability density function (*PDF*) for *y*. From the *PDF*, the mean value, $\hat{\text{y}}$ and the standard deviation, σ_y_, can be computed.

We note that the MC method captures non-linear effects and thus we cannot always expect direct agreement between $\sigma_{y}^{2}$ and FOFM-derived *u*^2^(*y*). Indeed, even if a bio-optical model contains weak non-linearities and MC model input uncertainties are normally distributed, the number of MC iterations still needs to be suitably large for $\sigma_{y}^{2}$ to agree with *u*^2^(*y*).

### Evaluation *R*_*rs*_ Dataset

To evaluate our FOFM uncertainty method, we used a dataset of high quality hyperspectral *R*_*rs*,*i*_ spectra (*N* = 1124). Hyperspectral radiometric measurements were collected *in situ* during three different expeditions, representing a range of oligotrophic to mesotrophic waters: the SABOR experiment in the Gulf of Maine/North Atlantic/Mid-Atlantic coast (July–August 2014); AE1319 in the North Atlantic and Labrador Sea (August–September 2013); and NH1418 in the Equatorial Pacific (September–October 2014). A HyperOCR system (Sea-Bird Scientific) deployed on a tethered profiler in “buoy mode” was used to collect upwelling radiance, *L*_*u*,*i*_ (W m^−2^ µm^−1^ sr^−1^), and downwelling irradiance, *E*_*d*,*i*_ (W m^−2^), spectra during deployments lasting ∼5 min. During sample collection, the instrument was allowed to drift far enough from the boat to avoid the ship’s shadow.

The spectra were dark and tilt-corrected, and the upper and lower 25th percentile of the *E*_*d*,*i*_ spectra were removed from both *E*_*d*,*i*_ and *L*_*u*,*i*_. The mean of the remaining spectra was used in subsequent analysis, providing one spectrum per deployment, and with uncertainties calculated as the standard deviation of the same spectra used to calculate the mean (N.B. uncertainties here represent only the experimental portion of the uncertainties, and calibration bias has not been accounted for). The *L*_*u*,*I*_ measurements were extrapolated to and across the air-water interface to obtain the water-leaving radiance, *L*_*w*,*i*_ (W m^−2^ sr^−1^), which were then used to calculate remote-sensing reflectance (*R*_*rs*,*i*_), defined as:

(4) $$R_{rs,i}=\frac{L_{w,i}}{E_{d,i}}$$

**FIGURE 1 |:**
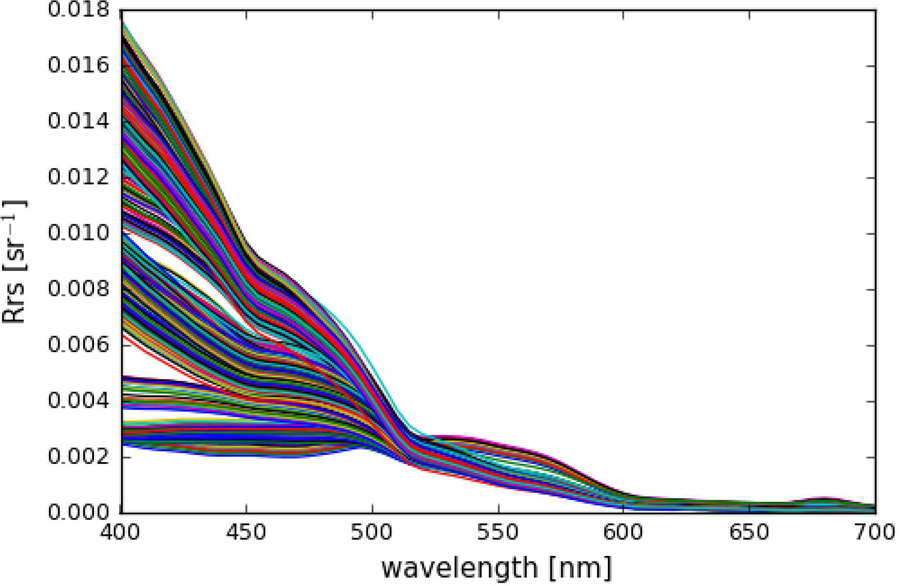
Hyperspectral remote-sensing reflectances (*N* = 1124) used in this study.

The spectra were additionally corrected for Raman scattering following methods in Westberry et al. (2013), which was necessary to compensate for the scattering that water molecules themselves can contribute to *L*_*w*,*i*_, especially at the blue wavelengths in very clear waters (McKinna et al., 2016). Finally, the *R*_*rs*_ spectra were normalized to remove the angular effect of the sun position in the sky relative to nadir, following methods in Lee et al. (2011). For a more detailed description of the *R*_*rs*,*I*_ calculations and processing, see Data and Methods section in Chase et al. (2017). All hyperspectral *R*_*rs*,*i*_ used in this study are shown in Figure 1.

Finally, each hyperspectral *R*_*rs*_ spectrum was spectrally sub-sampled. The resulting multiband *R*_*rs*,*i*_ dataset had sixteen 10 nm-wide spectral bands centered on: 412, 425, 443, 460, 475, 490, 510, 532, 555, 583, 617, 640, 655, 665, 678, and 710 nm. This multispectral subset spanned the visible domain and included bands from both past and present NASA sensors (e.g., SeaWiFS and MODIS).

### Radiometric Uncertainties

#### Spectrally Flat *R*_*rs*_ Uncertainties

For NASA ocean color bio-optical algorithms, model input quantities are typically remote sensing reflectances, *R*_*rs*,*i*_ (sr^−1^), which are derived from measured top-of-atmosphere radiances, *L*_*t*,*i*_ (W m^−2^ µm^−1^ sr^−1^), via atmospheric correction (AC) algorithms. Historically, a desirable science requirements for NASA ocean color missions has been *R*_*rs*,*I*_ with relative uncertainty of 5% (spectrally flat) or less (Hooker et al., 1992; Hooker and McClain, 2000; McClain et al., 2004; PACE Science Definition Team, 2018). Whilst not directly representative of a true sensor (see section Spectrally-Dependent *R*_*rs*_ Uncertainties), treating relative uncertainties in *R*_*rs*,*i*_ as spectrally flat is still useful under circumstances where detailed knowledge of sensor performance characteristics is limited, such as during pre-launch scoping studies, to provide rudimentary uncertainty estimates. In this study we first consider 5% relative uncertainty in *R*_*rs*,*I*_ to compare FOFM-to-MC calculations. We next use the FOFM method consider how spectrally flat relative uncertainties in *R*_*rs*_ of 1, 5, and 10% impact estimated OC and IOP uncertainties. Note, we treat spectrally flat relative uncertainties in *R*_*rs*_ of 1, 5, and 10% as spectrally uncorrelated.

#### Spectrally-Dependent *R*_*rs*_ Uncertainties

**FIGURE 2 |:**
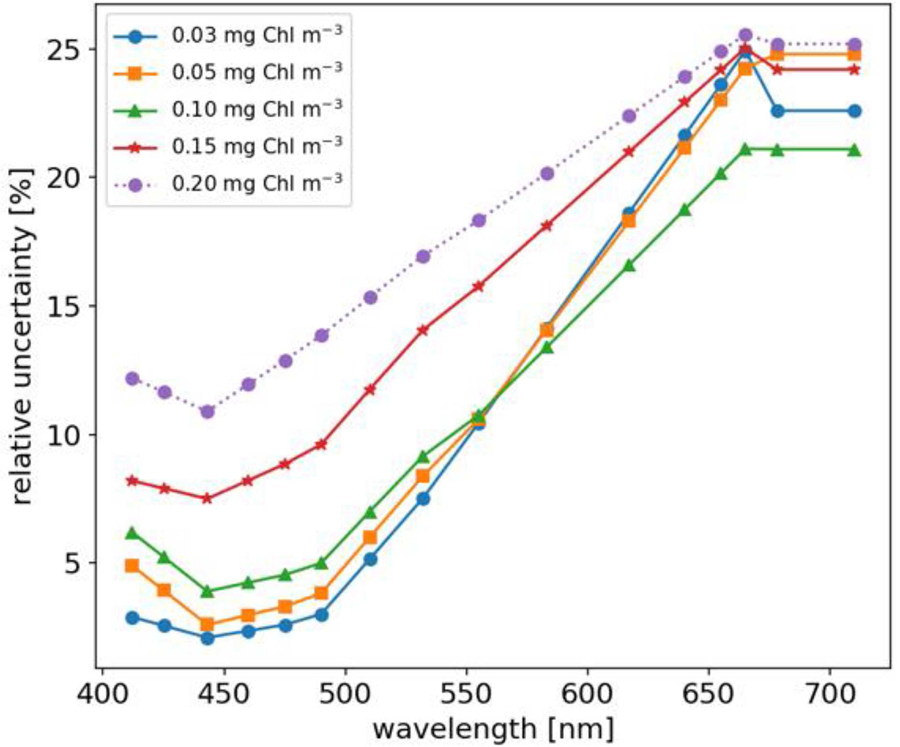
Relative uncertainties of *R*_*rs*_ varying with Chl concentration. Original data taken from Hu et al. (2013) and interpolated to the multispectral resolution used in this study.

We note that on-orbit uncertainties in *L*_*t*,*i*_ and *R*_*rs*,*i*_ have previously been quantified for NASA’s SeaWiFS and MODISA missions (Eplee et al., 2007; Hu et al., 2012a, 2013; Angal et al., 2015). Whilst historically 5% has been the desired accuracy goal for *R*_*rs*_ in the blue-green spectral range, work by Hu et al. (2013) reported that relative uncertainties of *R*_*rs*,*i*_ for SeaWiFS and MODISA increase monotonically with wavelength, and that *R*_*rs*,*i*_ relative uncertainty also varies as a function of *Chl*, or water-column optical complexity. To extend this study beyond spectrally flat relative uncertainties, we utilized the relative uncertainties for MODISA *R*_*rs*,*i*_ estimated for the North Atlantic Ocean (see Table 2 of Hu et al., 2013). To estimate relative uncertainty for a given *R*_*rs*,*i*_ spectra, we followed three steps: (i) linearly interpolate tabulated relative uncertainties to match the spectral resolution of our *in situ R*_*rs*,*i*_ dataset, (ii) estimate *Chl* concentration using NASA’s standard OC algorithm, and (iii) linearly interpolate the spectrally tabulated relative uncertainties to estimate relative uncertainty for observed *R*_*rs*,*i*_ based on the respective *Chl* concentration. Note, where estimated *Chl* exceeded 0.2 mg m^−3^ [beyond values reported by Hu et al. (2013)] we linearly extrapolated tabulated relative uncertainties. Figure 2 shows the spectral relative uncertainties in *R*_*rs*,*i*_ [*sensu* Hu et al. (2013)] used in this study and how they vary with *Chl* concentration. Note, spectrally-dependent relative uncertainties in *R*_*rs*_ computed as a function of *Chl* were treated as spectrally uncorrelated.

#### Spectrally-Correlated *R*_*rs*_ Uncertainties

Our initial analyses treated *R*_*rs*_ spectral uncertainties as uncorrelated, which in practice is an oversimplification. Indeed, AC algorithms utilize near-infrared bands to make assumptions about the contribution of atmospheric aerosols to *L*_*t*_ (Gordon and Wang, 1994; Bailey et al., 2010). Thus, *R*_*rs*,*i*_ uncertainties are inherently spectrally correlated. While much work has been done to characterize radiometric uncertainties of NASA sensors used for ocean color (Eplee et al., 2007; Hu et al., 2012a, 2013), few studies have quantified off-diagonal elements of the variance-covariance matrices for top-of-atmosphere radiance, **V**_*Lt*_, and remote sensing reflectances, **V**_*Rrs*_, respectively. We note that while beyond the scope of this work, parallel efforts are underway by the research community to derive pixel-by-pixel estimates of *u*(*R*_*rs*,*i*_) by propagating radiometric uncertainties through ocean color atmospheric correction algorithms (Gillis et al., 2018).

Recently, Lamquin et al. (2013) demonstrated a methodology to estimate **V**_*Lt*_ for MERIS data and propagate these through ESA’s clear water branch AC algorithm and into bio-optical data products. Critically, Lamquin et al. (2013) demonstrated that ignoring covariances can lead to overestimated data product uncertainties. In this study, using a similar methodology to Lamquin et al. (2013), we estimate **V**_*Lt*_ for SeaWiFS and then using a numerical approximation estimate **V**_*Rrs*_. A full description of this method can be found in Appendix F (Supplementary Material). We note that while our estimates of **V**_*Rrs*_ are somewhat rudimentary, they are still useful for demonstrating the importance of including covariance terms in FOFM-based uncertainty estimates.

### Satellite Data Processing

A SeaWiFS image of Hawaii captured on 1 December 2000 was used to demonstrate the FOFM methodology when applied to ocean color imagery. SeaWiFS Level-1 data was downloaded from NASA’s Ocean Biology Distributed Active Archive Center (NASA OB.DAAC) Level 1 and 2 Browser website (https://oceancolor.gsfc.nasa.gov/)^1^. Data were then processed from Level 1 to Level 2 using NASA Ocean Color Science Software (OCSSW). These processing steps include radiometric calibration, geolocation, and atmospheric correction. A prototype version of OCSSW code was used to compute *u*(*Chl*) using FOFM methodology where *u*(*R*_*rs*,*i*_) was estimated using an empirical methodology described in Appendix F (Supplementary Material).

## RESULTS

### Appraisal of Methodology

The MC methodology, while computationally expensive, was expected to give robust estimates of measurand uncertainties. Thus, MC outputs provided a benchmark to which the FOFM uncertainty estimates could be compared with for correctness. Direct calculations of FOFM uncertainties, *u*(*y*), were compared with MC output uncertainties, σ_y_. To compare MC and FOFM calculations we used 5% spectrally flat relative uncertainty in *R*_*rs*_ and computed the following comparison statistics: bias and Type II linear regression slope. When computing these statistics for the purpose of FOFM-to-MC comparisons, we assume that MC-estimated uncertainties were quasi-truth. We note that variables were log-transformed for these calculations following Seegers et al. (2018). Bias was computed as:

(5) $$bias=10^{\wedge }\left\{\sum_{k=1}^{N}\frac{\log_{10}\left(MC_{K}\right)-\log_{10}\left(FOFM_{k}\right)}{N}\right\},$$

where *N* = 1124 is the number of input spectra. Given that bias was computed using log-transformed variables, it becomes interpretable as multiplicative metrics (Seegers et al., 2018). We note that our bias calculations assume estimated OC and IOP uncertainties follow log-normal distributions; a property that is demonstrated later in the paper (e.g., Figures 4, 5).

**FIGURE 3 |:**
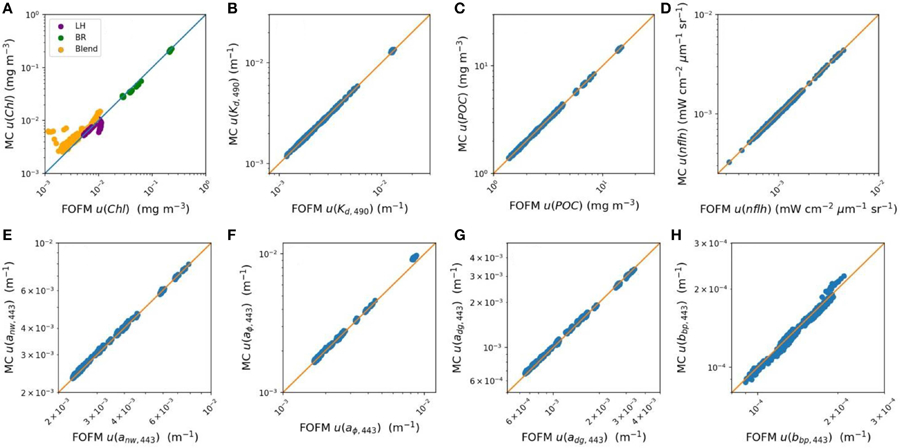
Scatter plot comparisons of data product uncertainties estimated from FOFM with those estimated from Monte Carlo (MC) simulations. **(A–D)** OC products Chl, *K*_*d,490*_, POC, and nflh, respectively. Note that the scatter plot of Chl uncertainty is color coded with respect to the method use to derive the output product (line height: purple, band ratio: green, blended: yellow). **(E–H)** IOP products *a*_*nw,443*_, *a*_*ϕ,443*_, *a*_*dg,443*_, and *b*_*bp,443*_, respectively.

The MC and FOFM estimation of derived product uncertainties were in good agreement for the following OC products: *K*_*d*,490_, *POC*, and *nflh*. This was indicated by slope and bias and statistics (Table 2) having values of, or near to, 1.0. However, regression statistics indicated *Chl* uncertainties derived using the FOFM method did not completely agree with the MC method (Table 2). To assess this discrepancy more closely, uncertainties in each component of the *Chl* algorithm were inspected, namely the band ratio (*BR*), line height (*LH*), and blended components. Regression statistics indicated that FOFM estimates of *Chl*_*blend*_ product uncertainties did not agree well with MC values and were typically biased low by 27%, visualized further by the color-coded scatter plot in Figure 3A.

Derived uncertainties for IOP products generally agreed with MC simulations. Specifically, Table 2 shows FOFM estimates of uncertainties with respect to MC estimates for *a*_*nw*,443_, *a*_*ϕ*,443_, *a*_*dg*,443_, and *b*_*bp*,443_ were biased low by 1%, low by 2%, low by 2% and, high by 2%, respectively. Slight disagreement between MC and FOFM estimates of *u*(*b*_*bp*,443_) can be visualized in Figure 3 when *u*(*b*_*bp*,443_) > 2.0 × 10^−3^ m^−1^. In addition, MC and FOFM estimates of *u*(*a*_*ϕ*,443_) showed slight disagreement when *u*(*a*_*ϕ*,443_) > 1.0 × 10^−2^ m^−1^.

These results demonstrate that while FOFM uncertainty calculations are computationally inexpensive, they serve as approximations only, especially in the case of *Chl*. Indeed, while FOFM-derived uncertainties can be expected to agree with MC-derived values for simple functions that vary linearly, it may not be unusual for FOFM-derived uncertainties to differ from MC-derived values; particularly when analyzing complicated non-linear problems (Putko et al., 2001; Mekid and Vaja, 2008). For example, with the IOPs we found slight differences in the order of 1% between MC and FOFM uncertainty estimates. For such mathematical functions, higher order methods such as Second Order First Moment (SOFM) methods may be useful, however, the added mathematical complexity may be prohibitive.

### Uncertainties Estimated From *in situ* Radiometric Data

#### OC Product Uncertainties

**FIGURE 4 |:**
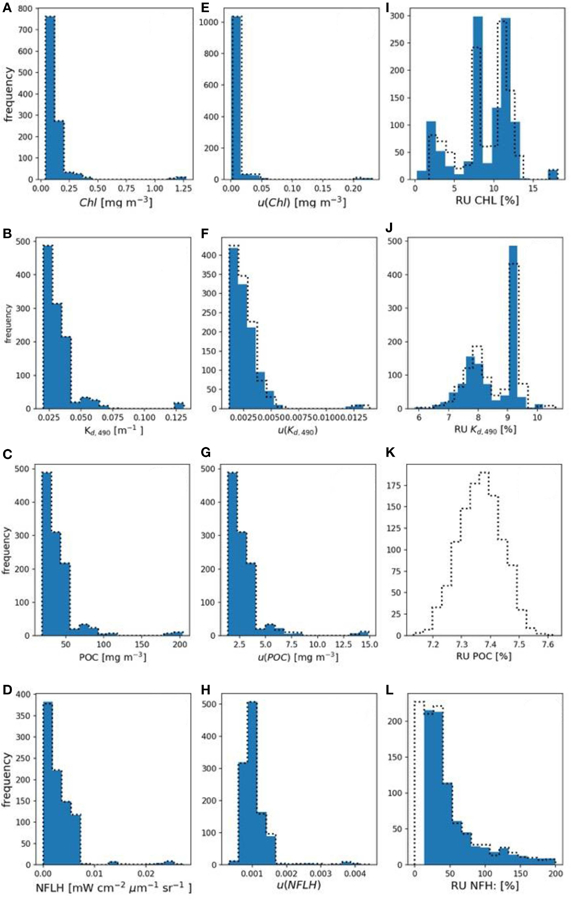
**(A–D)** Histograms of derived Chl, *K*_*d,490*_, POC, and nflh, respectively. **(E–H)** Histograms of FOFM-estimated uncertainties in derived Chl, *K*_*d,490*_, POC, and nflh, respectively computed using 5% spectrally flat, uncorrelated uncertainty in input *R*_*rs*_. **(I–L)** Histograms of FOFM-estimated relative uncertainties in derived Chl, *K*_*d,490*_, POC, and nflh, respectively. Note: FOFM-estimates of POC relative uncertainties in this example were invariant. Dashed curves represent MC results, solid blue bars represent FOFM results.

Using the multispectral *R*_*rs*_ evaluation dataset, uncertainties in derived OC products associated with 5% spectrally-flat relative, uncorrelated uncertainty in *R*_rs_ were computed. Figure 4 shows histograms of derived OC products, absolute uncertainties, and relative uncertainties. MC computations are summarized in Table 3, while FOFM computations are provided for comparative purposes in Table 4.

The range of derived *Chl* confirmed that the dataset spans oligotrophic (0.04 mg m^−3^) to mesotrophic conditions (1.28 mg m^−3^) with a median value of 0.11 mg m^−3^. Values of *u*(*Chl*) span four orders of magnitude and have median values of 7.00 × 10^−3^ and 6.70 × 10^−3^ mg m^−3^ for the MC and FOFM methods, respectively. The relative uncertainties for *Chl* span a single order of magnitude and have median values of 9.74 and 9.67% for the MC and FOFM methods, respectively. Although the histogram of derived *Chl* in Figure 4 appears log-normally distributed, two distinct peaks are present; a low peak (ranging from 0 to 0.5 mg m^−3^) and a high peak (centered on 1.1 mg m^−3^). Since bio-optical properties are log-normally distributed in the ocean (Campbell, 1995), the peaks observed in the distributions of derived bio-optical variables are probably due to the limited size of the hyperspectral *R*_*rs*_ dataset (*N* = 1124), that does not uniformly span the entire range of oceanic conditions (see Figure 1A in Chase et al., 2017).

The range of derived *K*_*d,490*_ spans an order of magnitude with a median value of 0.0291 m^−1^. The values of *u*(*K*_*d,490*_) also span an order of magnitude with median values of 2.68 × 10^−3^ m^−1^ for both MC and FOFM calculations. The relative uncertainties for *K*_*d,490*_ span a single order of magnitude and have a median value of 8.94 and 8.91% for MC and FOFM calculations, respectively. The range of derived *POC* spans two orders of magnitude with a median value of 33.1 mg m^−3^. The values of *u*(*POC*) span an order of magnitude and have median values of 2.44 and 2.42 mg m^−3^ for MC and FOFM calculations, respectively. The relative uncertainties in *POC* have a value of 7.37 and 7.31% for MC and FOFM calculations, respectively. We note that the relative uncertainty in *POC* as computed by FOFM method exhibits no spread. For uncorrelated, spectrally flat relative uncertainties, *u*(*POC*)/*POC* is a function of *u*(*R*_*rs,443*_)/*R*_*rs,443*_ and *u*(*R*_*rs,555*_)/*R*_*rs,555*_. Thus, when *u*(*R*_*rs,443*_)/*R*_*rs,443*_ and *u*(*R*_*rs,555*_)/*R*_*rs,555*_ are fixed (e.g., at 5%), *u*(*POC*)/*POC* is fixed. In practice, this will not always hold true, particularly when relative uncertainties in *R*_*rs*_ are variable and spectrally dependent. We note that in Figure 4 the MC-derived relative uncertainties for *POC* are normally distributed over a narrow range centered on 7.37%.

The range of *nflh* spans three orders of magnitude with an MC-estimated median value of 2.20 × 10^−3^ mW cm^−2^ µm^−1^ sr^−1^. We note that direct calculations of *nflh* resulted in a median value of 2.19 × 10^−3^ mW cm^−2^ µm^−1^ sr^−1^. The values of *u*(*nflh*) span an order of magnitude with median values of 9.86 × 10^−4^ and 9.87 × 10^−4^ mW cm^−2^ µm^−1^ sr^−1^ for MC and FOFM calculations, respectively. The median relative uncertainty in *nflh* was 41.9 and 42.1% for MC and FOFM calculations, respectively (Figure 4). We note that the range of relative errors for *nflh* is very large (for MC calculations: 14.8–1.7 × 10^4^%), and these should be interpreted with a caution. Low values of *nflh*, in the order of 1 × 10^−6^ mW cm^−2^ µm^−1^ sr^−1^, were derived from the evaluation dataset which in most likelihood would be beyond the detection limit of existing ocean color sensors. Further, while the absolute uncertainties associated with these low *nflh* values may also be small in magnitude, they can still manifest as large relative uncertainties.

#### IOP Product Uncertainties

**FIGURE 5 |:**
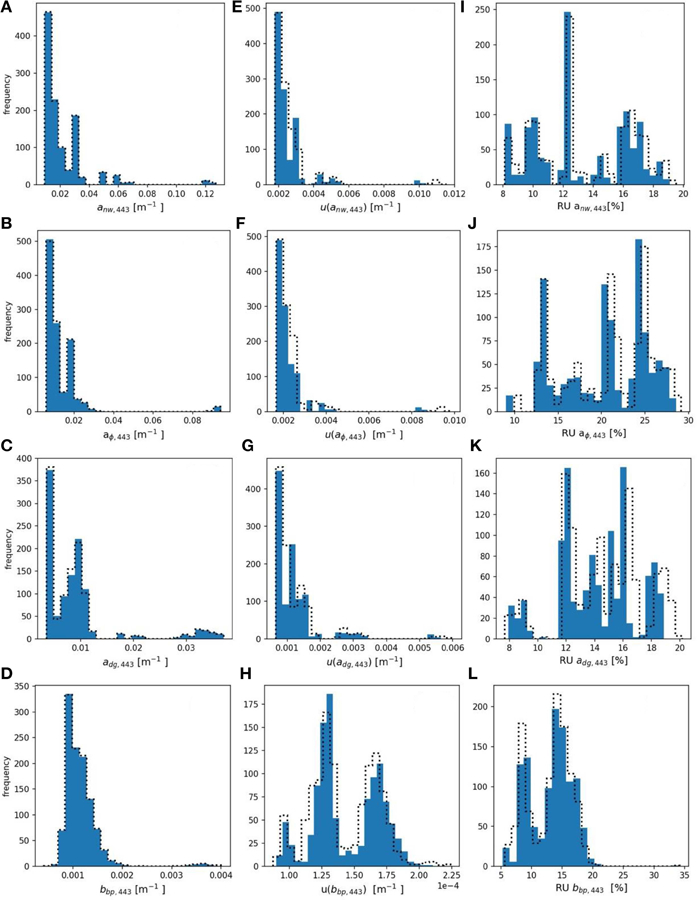
**(A–D)** histograms of derived *a*_*nw,443*_, *a*_*dg,443*_, *a*_*ϕ*,*443*_, and *b*_*bp,443*_, respectively. **(E–H)** histograms of FOFM-estimated uncertainties in derived *a*_*nw*,*443*_, *a*_*dg*,*443*_, *a*_*ϕ*_,*443*, and *b*_*bp*,*443*_, respectively, computed using 5% spectrally flat, uncorrelated uncertainty in input *R*_*rs*_. **(I–L)** histograms of FOFM-estimated relative uncertainties in derived *a*_*nw.443*_, *a*_*dg,443*_, *a*_*ϕ*_, *443*, and *b*_*bp,443*_, respectively. Dashed curves represent MC results, solid blue bars represent FOFM results.

Using the radiometric evaluation dataset, uncertainties in derived IOP products associated with 5% relative, uncorrelated uncertainty in *R*_*rs*,*i*_ were computed following the methodology in Appendix E (Supplementary Material). Figure 5 shows histograms of derived IOP products, absolute uncertainties, and relative uncertainties. MC computations are summarized in Table 5 while FOFM computations are provided for comparative purposes in Table 6.

The range of derived *a*_*nw,443*_ spans two orders of magnitude with a median value of 0.0185 m^−1^. Values of *u*(*a*_*nw,443*_) span an order of magnitude with median values of 2.31 × 10^−3^ and 2.26 × 10^−3^ m^−1^ for MC and FOFM methods, respectively. The median relative uncertainty in *a*_*nw*,443_ spans a single order of magnitude and has median values of 12.6 and 12.2% for MC and FOFM methods, respectively. The range of *a*_*ϕ,*443_, *a*_*dg,443*_, and *b*_*bp,443*_ all span a single order of magnitude with median values of 9.6 × 10^−3^, 8.71 × 10^−3^, and 1.08 × 10^−3^ m^−1^, respectively. Absolute uncertainties in IOPs all span two orders of magnitude apart from *u*(*a*_*ϕ,443*_) which spanned a single order of magnitude. Highest relative uncertainties of all GIOP-derived products are for *a*_*ϕ,*443_ (∼20%), whereas *a*_*nw,440*_, *a*_*dg,440*_, and *b*_*bp,440*_ have relative uncertainties of similar magnitude that are all <15%.

#### Summary of MC and FOFM Comparisons

FOFM and MC estimates of OC and IOP uncertainties were generally in good agreement. This provides confidence that our FOFM analytical formulations were correct. However, FOFM-to-MC comparisons of *Chl* and IOP uncertainties, whilst similar in magnitude, exhibited a degree of scatter around the one-to-one line. We expect that these differences may be due to the MC method’s ability to handle non-linearity and discontinuities in the models more robustly than the FOFM approach. For example, the *Chl* model has several complex features such: switching between *Chl*_*BR*_ *and Chl*_*LH*_, the *Chl*_*BR*_ model’s selection of maximum band ratios, and the blending of *Chl*_*BR*_ *and Chl*_*LH*_, which may not be fully captured by the FOFM method.

We thus found FOFM-to-MC comparisons to be useful as a “quick acceptability checking” of FOFM calculations. In practice, however, one should not always assume the two methods will closely agree as the MC model may handle non-linearities and discontinuities more robustly than the FOFM method. The FOFM and MC calculations also indicate that for normally distributed radiometric input uncertainties, the estimated output uncertainties for OC and IOP were log-normally distributed (as per Figures 4, 5). Such highly dynamic and variable nature of uncertainties in ocean color data products highlights the need for these estimates to be done on a pixel-by-pixel basis, rather than a single scene-wide estimate, further justifying the need for simplified, computationally inexpensive approach (i.e., FOFM).

We note that our FOFM uncertainty formulation for the GIOP currently does not consider uncertainty in spectral shape models [i.e., $u\left(a_{\phi ,i}^{*}\right)$ and $u\left(b_{bp,i}^{*}\right)$]. Indeed, we believe that this may be why there were some noticeable differences when comparing FOFM and MC methods, for example: when *u*(*b*_*bp,443*_) > 2.00 × 10^−4^ m^−1^ (Figure 3H). In a cursory study, we re-ran both FOFM and MC calculations with the shape models parametrized as spectral constants (i.e., having no uncertainties). This resulted in improved FOFM-to-MC comparisons (results not shown) and further highlighted how spectral shape uncertainties impact our FOFM uncertainty estimates. As part of future work, we thus plan to extend our current GIOP FOFM uncertainty formulation to include the spectral shape uncertainties. Additionally, we note that $u\left(a_{\phi ,i}^{*}\right)$ and $u\left(b_{bp,i}^{*}\right)$, computed as functions of *Chl* and a red-green *R*_*rs*,*i*_ ratio, respectively, are also correlated. Thus, an improved GIOP FOFM uncertainty formulation should also consider covariances between spectral shape models.

#### GIOP Model Misfit Uncertainties

In this analysis we used our high-quality evaluation *R*_*rs*_ dataset to approximate GIOP model misfit uncertainties. Our assumptions in this exercise were: (i) the uncertainties in our *R*_*rs*_ dataset are small, and (ii) the least squares residual of the optimal solution (model misfit) are thus due to an imperfect model.

In this analysis we first computed the error-covariance matrix, **E**_rrs_, for each *R*_*rs*_ observation as follows: (i) employ the Levenberg-Marquardt non-linear least squares optimization to iteratively find an optimal solution for the free variables *x*_*ϕ*_, *x*_*dg*_, and *x*_*p*_ which correspond to *Chl* concentration, *a*_*dg*,*440*_, and *b*_*bp*,*440*_, respectively (see Appendix E in Supplementary Material for further detail). We note that in the standard implementation of the GIOP, the cost function (Chi-squared) is unweighted. (ii) feed the optimal set of *x*_*ϕ*_, *x*_*dg*_, and *x*_*p*_ back in the forward reflectance model to compute a best-fit spectral sub-surface remote sensing reflectance, $r_{rs,i}^{\mathrm{mod}}$. (iii) calculate the spectral residual, ε_*rrs*,*i*_, between $r_{rs,i}^{\mathrm{mod}}$ and sensor-observed subsurface remote sensing reflectance. (iv) set the diagonal elements of **E**_rrs_ as the square of ε_*rrs*,*i*_.

Next, by substituting **E**_rrs_ for **V**_rrs_ in Equation E13 the parameter error-covariance matrix, **E**_x_, can be computed as:

(6) $$E_{x}=J^{-1}E_{rrs}{\left(J^{\text{T}}\right)}^{-1}$$

Where **J** is the Jacobian matrix of the forward model (see Appendix E in Supplementary Material for derivation). Finally, the estimates of parameter uncertainties due to model misfit were calculated as the square root of the diagonal elements of **E**_x_. The model-misfit uncertainties are summarized in Table 7 and compared to parameter uncertainties due to Hu spectrally-dependent radiometric uncertainties (as per Table 6).

We found that estimated GIOP model misfit uncertainties were 60–90% smaller than those imparted by radiometric uncertainty. Thus, by combining the two during pixel-by-pixel processing, it would be possible to more completely estimate *u*_*measurement*_(*y*) for IOPs. However, we accept that our FOFM model-data misfit approach is approximate only and does not consider all uncertainties in the GIOP model formulation.

### Comparing Product Uncertainties Due to Various Radiometric Input Uncertainties

**FIGURE 6 |:**
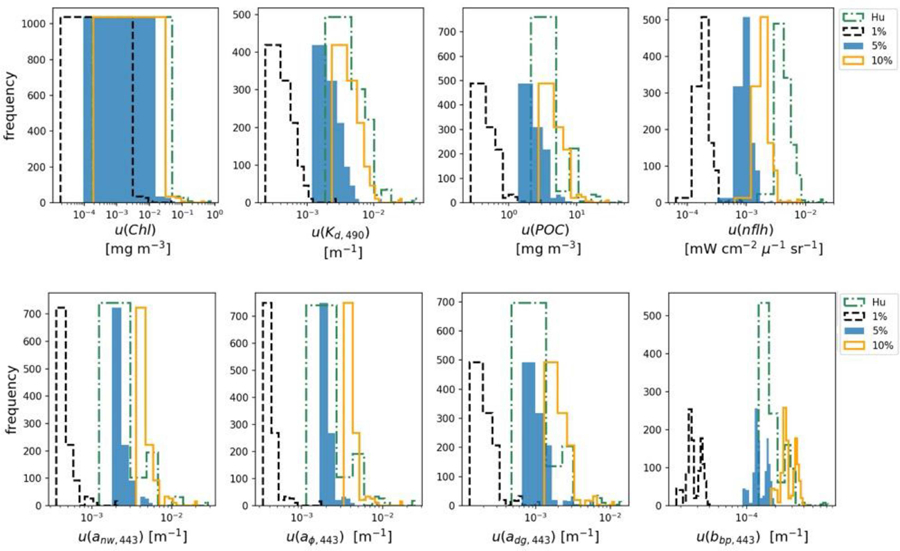
Upper row are histograms of derived OC data products uncertainties estimated using the FOFM method. Bottom row are histograms of derived IOP data product uncertainties estimated using the FOFM method. The four histograms in each subplot correspond to four different input u(*R*_*rs*_): spectrally flat *R*_*rs*_ relative uncertainties of 1% (dashed black), 5% (blue), and 10% (orange) as well as spectrally dependent relative uncertainties taken from Hu et al. (2013) outlined in green dashed line.

In order to evaluate the impact of different *R*_*rs*_ uncertainty values on derived product uncertainties, using the FOFM method we: (i) propagated spectrally flat, uncorrelated *R*_*rs*_ relative uncertainties of 1, 5, and 10% through OC and IOP models, and (ii) propagated spectrally-dependent, uncorrelated *u*(*R*_*rs*_) through OC and IOP models by linearly interpolating/extrapolating tabulated data published by Hu et al. (2013), referred to as “Hu uncertainties” (see Figure 2). Summary results of this analysis are given in Tables 8, 9. As expected, introducing spectrally flat, uncorrelated *R*_*rs*_ uncertainties of lower and higher value than the previously evaluated 5%, resulted in respectively, lower and higher uncertainties in data products, while the distribution of uncertainties kept the same shape as for the 5% run (Figure 6). For the product uncertainties derived using the “Hu *R*_*rs*_ uncertainties,” both the shape of the distribution and median values changed from the 5% run (Figure 6). These results demonstrate the importance of considering spectral dependence in radiometric uncertainties. Notably, considering spectrally flat 5% relative uncertainties in *R*_*rs*_ for a data product such as *nflh*, which utilizes red-end bands, may result in significant underestimation of likely data product uncertainties.

Spectrally flat relative uncertainty in *R*_*rs*_ (e.g., 5% in the blue-green region) is a commonly used accuracy goal for ocean color missions. However, we know from on-orbit data that sensors such as SeaWiFS and MODIS have largely not achieved their desired accuracy goals over the full spectral range (Hu et al., 2013), particularly at red wavelengths. In lieu of any knowledge of a sensor’s radiometric uncertainty characteristics (e.g., during design trade studies), one might decide to utilize desired relative radiometric accuracy goals to approximate ocean color data product uncertainties. However, our results have shown spectrally flat (5%) and spectrally-dependent (Hu) relative *R*_*rs*_ uncertainties lead to different estimates of OC and IOP uncertainties. Indeed, for improved uncertainty estimates, we recommend the use of more representative spectrally-dependent *u*(*R*_*rs*_)/*R*_*rs*_, if known.

### Application to Satellite Chlorophyll Image

**FIGURE 7 |:**
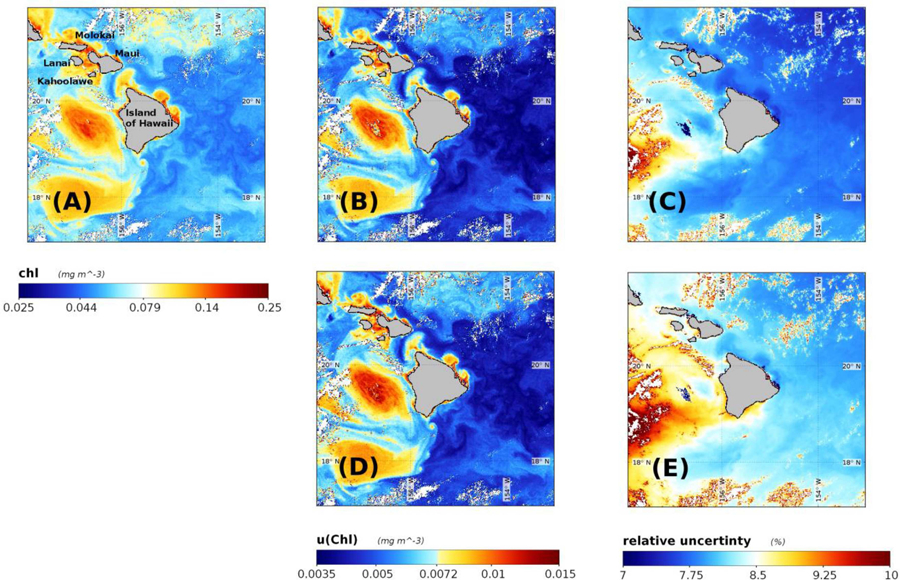
Derived data products for a SeaWiFS image of waters surrounding the Hawaii Islands captured on 1 December 2000. **(A)** Chl concentration derived using OCI algorithm, **(B)** u(Chl) computed with covariances included, **(C)** relative uncertainty in Chl computed with estimated *R*_*rs*_ covariances included, **(D)** u(Chl) calculated without estimated *R*_*rs*_ covariances included, and **(E)** relative uncertainty in Chl computed without estimated *R*_*rs*_ covariances included.

The potential impact that spectrally-correlated uncertainties in *R*_*rs*_ have upon ocean color data product uncertainties was evaluated using a scene of the southern Hawaiian Islands captured on 1 December 2000 (Figure 7). We have estimated on a pixel-by-pixel basis the covariance matrix of remote sensing reflectances, **V**_Rrs_, as per the methodology described in Appendix F (Supplementary Material). Estimates of *u*(*Chl*) were then calculated both with- and without the off-diagonal terms in **V**_Rrs_ to demonstrate the impact of incorporating covariance terms (if known) when estimating uncertainties.

The sample SeaWiFS *Chl* image (Figure 7A) shows that the clearest waters occurred southeast of Island of Hawaii (largest island) with two large eddies to the west. Regions of elevated *Chl* concentration are also visible along the northeast coast of the Island of Hawaii, and also adjacent to coastal waters of four islands (Maui, Lanai, Molokai, and Kahoolawe) to the northwest of Hawaii. Derived *Chl*_*blend*_ ranges from 1.83 × 10^−3^ to 0.498 mg m^−3^ with a median of 0.066 mg m^−3^. When the off-diagonal terms in **V**_Rrs_ were considered, the estimated values of *u*(*Chl*) ranged from 1.30 × 10^−3^ to 0.075 mg m^−3^ with a scene-wide median of 5.20 × 10^−3^ mg m^−3^ (Figure 7B) and the relative uncertainties spanned 0.84–38.6% with a median of 7.89% (Figure 7C). When the off-diagonal terms in **V**_Rrs_ were not considered (i.e., set to zero), estimated values of *u*(*Chl*) ranged from 1.30 × 10^−3^ to 0.109 mg m^−3^ with a scene-wide median of 5.50 × 10^−3^ mg m^−3^ (Figure 7D) and relative uncertainties spanning 0.85–46.1 % with a median of 8.27% (Figure 7E). Note, these image statistics were computed with standard NASA level-2 quality control flags applied to remove the effect of: land, clouds, sun glint, atmospheric correction failure, product failure, and straylight contamination.

These results demonstrate how a FOFM method can be utilized in operational processing code to estimate uncertainties in derived bio-optical data products. The FOFM method was straightforward to implement within l2gen code and did not add any appreciable processing overhead. Whilst our estimation of **V**_Rrs_ is rudimentary (Appendix F in Supplementary Material), it allowed us to consider the covariance terms in the FOFM derivation of *u*(*Chl*). Critically, we demonstrated that the inclusion of off-diagonal covariance terms from **V**_Rrs_ led to lower estimates of both *u*(*Chl*) and *u*(*Chl*)/*Chl* when compared to the same calculations performed with off-diagonal elements of **V**_Rrs_ set to zero; a result consistent with findings of Lamquin et al. (2013). Additionally, this example was done with an operational processing code, demonstrating the easiness of implementing a FOFM method within day-to-day ocean color processing.

### POC Algorithm Case Study

Recall from Equation 1, we broadly defined measurement uncertainty as having two sources: data uncertainty and model uncertainty. Throughout this paper we have focused heavily on deriving data uncertainties (i.e., propagation of radiometric uncertainty) which is useful if one is trying understand how a specific sensor’s noise characteristics may impact derived data product uncertainties. However, this information alone does not provide a complete picture of measurement uncertainty; model uncertainty also needs to be considered. We thus wish to demonstrate how with knowledge of model uncertainties one can draw more complete conclusions about biogeochemically-relevant data product uncertainties. As such, we present a case study in which we estimate *POC* measurement uncertainty for two different algorithms: (i) Stramski et al. (2008a) and (ii) Rasse et al. (2017).

Our motivation here is to solely demonstrate how one might develop algorithm uncertainty budgets (data and model uncertainty as per Equation 1) using a FOFM framework. Our calculations, however, are limited by: (i) the representativeness of our *in situ R*_*rs*_ dataset which does not encompass all optical water-types found in the World’s oceans, (ii) our spectral *u*(*R*_*rs*_) values which are estimated from data published by Hu et al. (2013) for a MODIS-like sensor without co-variance terms, and (iii) our knowledge of model uncertainties, such as coefficients uncertainties, which is limited to those reported in literature and/or our best-guess estimates. We hence caution the reader should not use our reported numbers as a basis for algorithm selection.

#### POC Measurement Uncertainty Estimates

In this exercise, we performed rudimentary calculations to estimate measurement uncertainty budgets for two *POC* algorithms: (i) NASA’s standard *POC* algorithm (Stramski et al., 2008a) and (ii) the IOP-based model of Rasse et al. (2017). Conveniently for this exercise, both *POC* models have a power law formulation:

(7) $$POC=a_{poc}X^{b_{poc}}$$

where *X* in Stramski et al. (2008a) is a blue-to-green reflectance ratio (*R*_*rs*,443_/*R*_*rs*,*555*_, as per Appendix C in Supplementary Material) and the coefficients *a*_*poc*_ and *b*_*poc*_ have the values of 203.2 and −1.034, respectively. For the approach of Rasse et al. (2017) *X* is *b*_*bp*_,_*470*_ and the coefficients *a*_*poc*_ and *b*_*poc*_ have the values of 141,253 and 1.18, respectively. Note, in this case study we use GIOP-derived estimates of *b*_*bp*_,_*470*_ as inputs to the Rasse et al. (2017) model.

First, let us consider the model uncertainty component due to imperfect model coefficients. For both *POC* algorithms, with the coefficients *a*_*poc*_ and *b*_*poc*_ and their assigned uncertainties of *u*_*model*_(*a*_*poc*_) and *u*_*model*_(*b*_*poc*_), respectively, we can estimate the model variance for *POC* as:

(8) $$u_{\text{model}}^{2}\left(POC\right)={\left(X^{b_{poc}}\right)}^{2}u_{\text{model}}^{2}\left(a_{poc}\right)\;\;\;\;\;\;\;+{\left(a_{poc}X^{b_{poc}}\log\left(X\right)\right)}^{2}u_{\text{model}}^{2}\left(b_{poc}\right)\;\;\;\;\;\;\;+{\left(a_{poc}b_{poc}X^{b_{poc}-1}\right)}^{2}u_{\text{model}}^{2}\left(X\right)$$

In the third term on the right-hand side of Equation 8, we set *u*_*model*_(*X*) = 0 and *u*_*model*_(*X*) = *u*_*model*_(*b*_*bp*,*470*_) for Stramski et al. (2008a) and Rasse et al. (2017), respectively. We have also assumed the covariance of the coefficients *a*_*poc*_ and *b*_*poc*_, which are determined by regression fit, is zero. For the Rasse et al. (2017) model, the reported model coefficient uncertainties *u*_*model*_(*a*_*poc*_) and *u*_*model*_(*b*_poc_) are 45,534 and 0.046, respectively. For the Stramski et al. (2008a) model, values of *u*_*model*_(*a*_*poc*_) and *u*_*model*_(*b*_poc_) were not reported. We did, however, estimate these model uncertainties by reanalyzing the original published dataset (Stramski et al., 2008b) and considering the likely uncertainty introduced by not accounting for the effect of filter pad absorption of *POC* (Novak et al., 2018). Following this cursory analysis (results not shown), we estimated *u*_*model*_(*a*_*poc*_) and *u*_*model*_(*b*_poc_) for the Stramski et al. (2008a) model to be ∼2.20 and 0.015, respectively.

Next, we considered the data uncertainty component. The Stramski et al. (2008a) model’s data uncertainty FOFM calculus was formulated in Appendix C (Supplementary Material). For the Rasse et al. (2017) model, we first estimated *u*_*data*_(*b*_*bp*_,_*470*_). To do so, *b*_*bp*_,_*470*_ was calculated from GIOP-derived *b*_*bp*,*440*_ as:

(9) $$b_{bp,470}=b_{bp,440}\times {\left(\frac{440}{470}\right)}^{\gamma }$$

The variance in *b*_*bp*_,_470_ due to data uncertainty was then estimated as:

(10) $$u_{data}^{2}\left(b_{bp,470}\right)={\left(\frac{\partial b_{bp,470}}{\partial b_{bp,440}}\right)}^{2}u_{data}^{2}\left(b_{bp,440}\right)+{\left(\frac{\partial b_{bp,470}}{\partial \gamma }\right)}^{2}u_{data}^{2}\left(\gamma \right)\;\;\;\;\;\;\;+2\frac{\partial b_{bp,470}}{\partial b_{bp,440}}\frac{\partial b_{bp,470}}{\partial \gamma }u_{data}^{2}\left(b_{bp,440},\gamma \right)$$

For this exercise, we used GIOP-derived values of *u*_*data*_(*b*_*bp*_,_470_) and *u*(γ). The correlation between derived values of *b*_*bp*_,547 and γ was used to estimate the covariance term *u*(*b*_*bp*_,_547,_ γ) as −1.64 × 10^−6^ m^−1^ nm^−1^. Using, the GUM methodology the variance in the Rasse et al. (2017) *POC* model due to data uncertainty was then estimated as:

(11) $$u_{data}^{2}\left(POC\right)={\left(a_{poc}b_{poc}{\left(b_{bp,470}\right)}^{b_{poc}-1}\right)}^{2}u_{data}^{2}\left(b_{bp,470}\right)$$

We finally estimated the measurement uncertainty budgets for both *POC* models using our *R*_*rs*_ evaluation dataset and with Hu spectrally-dependent, uncorrelated radiometric uncertainties (results are shown in Table 10).

In our rudimentary measurement uncertainty budget for the Stramski et al. (2008a) *POC* algorithm, we found the contribution of data (radiometric) uncertainty was larger than model uncertainty. Conversely, for the Rasse et al. (2017) *POC* algorithm, the contribution of model uncertainty was larger than data uncertainty. Whilst these *POC* algorithm uncertainty budgets may not be fully representative due to the assumptions we partook here, the exercise nonetheless demonstrates an important point: data and model uncertainties should both be considered if one wishes to use uncertainties as a means of benchmarking/comparing ocean color algorithms.

**FIGURE 8 |:**
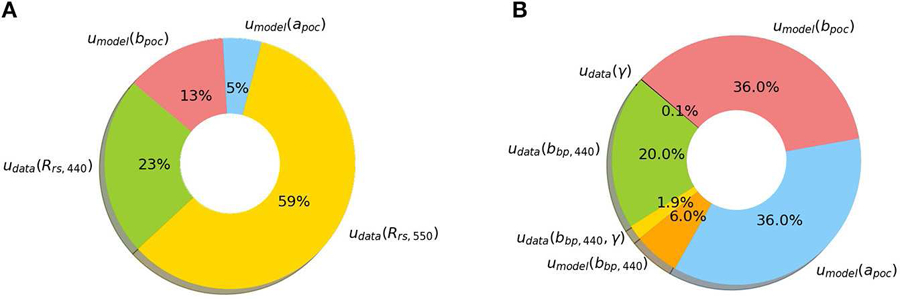
Pie charts demonstrate how individual uncertainty sources contribute to estimates of total measurement uncertainty. Here we consider: **(A)** a blue-green band-ratio *POC* algorithm and **(B)** an IOP-based *POC* algorithm. We note that these examples are intended to illustrate how one might visualize source contributions to measurement uncertainty. These plots are not intended for algorithm comparison purposes.

From an algorithm development perspective one can also use FOFM method to explore the relative contribution of individual uncertainty sources to the combined measurement uncertainty. We have graphically displayed the estimated component uncertainty contribution for each *POC* algorithm using pie charts (Figure 8). Such information may assist algorithm designers identify and minimize uncertainty sources within a model.

#### Summary of POC Case Study

Our brief example demonstrates the benefits of using the FOFM method for analytically estimating measurement uncertainty in *POC*. From an ecological perspective, this is particularly useful if one is trying to understand the variability in observed patterns, and distinguish real change from variation in uncertainty. Additionally, it allows for sensitivity analysis, thereby providing a guideline for improving model parameterization. The case study demonstrates how an uncertainty budget can provide additional information to end-users regarding data product quality, potentially informing algorithm selection, and/or guiding new algorithm development. Although ocean color algorithms are typically benchmarked based upon validation matchup metrics (Seegers et al., 2018), we expect model selection and development may be better guided by considering how data and model uncertainties manifest in derived data products.

This case study highlights a challenge if one wishes to compare/benchmark legacy ocean color algorithms based on their measurement uncertainty; one must have reasonable and complete knowledge of both data and model uncertainties to do so. Whilst we have demonstrated that it is possible to estimate and propagate random radiometric uncertainties using the FOFM framework, estimating model uncertainties remains a challenge. This is because model component uncertainties (e.g., model coefficient uncertainties) of legacy ocean color algorithms were not routinely reported. To address this, re-analysis of the structure of legacy ocean color algorithms using high quality bio-optical datasets, such as NASA’s bio-Optical Marine Algorithm Dataset (NOMAD; Werdell and Bailey, 2005), may be necessary. Without such knowledge, it remains a challenge to formulate complete measurement uncertainty budgets for legacy ocean color algorithms.

## CONCLUSIONS

In this paper we demonstrated a FOFM-based method for estimating uncertainties in a selection of NASA OC and IOP products, namely: *Chl*, *K*_*d*,*490*_, *POC*, *nflh*, *a*_*nw*,*440*_, *a*_*ϕ*,440_, *a*_*dg*,*440*_, and *b*_*bp*,*440*_, due to sensor-observed radiometric uncertainty. Using a high quality hyperspectral *R*_*rs*_ dataset subsampled to our target wavelengths, we first appraised the FOFM methodology by comparing FOFM-derived uncertainty estimates with uncertainties estimated from MC simulations with an assumed relative spectrally flat, uncorrelated uncertainty in *R*_*rs*_ of 5%. Our analyses showed that OC and IOP uncertainties estimated using the FOFM method generally agreed with MC simulations. Collectively, the FOFM-to-MC comparisons provided a basis for checking the correctness of the FOFM formulations, which are often algebraically complex. Further, we demonstrated that the FOFM formulation, which is computationally inexpensive, can be applied in routine pixel-by-pixel data processing for estimating uncertainties in derived ocean color data products.

This paper has primarily focused on propagating radiometric uncertainties through bio-optical models (*u*_*data*_(*y*) in Equation 1). In practice, the combined measurement uncertainty in derived ocean color data products is expected to be larger once model uncertainties are included. In this study, we have broadly assumed that coefficients within the bio-optical algorithms themselves are errorless, which is not the case. Indeed, most coefficients in bio-optical algorithms have been derived empirically using *in situ* oceanographic datasets, which themselves have inherent uncertainties due to measurement method and environmental variability. The GIOP, for example, makes assumptions about spectral shapes of IOPs, utilizes an approximate forward reflectance model (Gordon et al., 1988), and employs a model to convert *R*_*rs*_,_*i*_ to *r*_*rs*,*i*_ (Lee et al., 2002). Thus, there are a number of GIOP model components whose uncertainties, if characterized, may improve the overall estimate of IOP measurement uncertainty. Our case study of *POC* algorithms also highlighted how the addition of model (e.g., coefficient) uncertainties can further inform end-users, and may potentially guide algorithm development and/or selection.

Although this work represents a first step toward implementing pixel-by-pixel uncertainty estimates in NASA operational ocean color processing code, we recognize that continued effort is required. For example, strategies for quantifying uncertainties in look-up-table (LUT) based models, such as the two-band particulate inorganic carbon (PIC) algorithm (Balch et al., 2005) and bidirectional reflectance distribution function (BRDF) correction (Morel et al., 2002), are needed. Globally, there are a multitude of ocean color algorithms maintained by various researchers and/or institutes and formulating uncertainty estimates must be a collective effort. While the community continues to innovate new bio-optical algorithms, we strongly encourage model developers to characterize uncertainties as a matter of routine.

As we enter the hyperspectral world of PACE, it is credible to expect an evolutionary leap in remote sensing observation of ocean processes detailing, for example, phytoplankton diversity, physiological preferences, and ecology from space. This, parallel to the increase in computational power of the day-to-day data processing, will allow for more complex algorithms; algorithms which will need detailed evaluation of uncertainty budgets, to understand what is real, and what is hidden under the dashed line.

## Supplementary Material

Appendices A-F

## Figures and Tables

**TABLE 1 |:** Bio-optical ocean color data products.

Product name	Product suite	Symbol	Units	References
Chlorophyll-a pigment concentration*	OC	*Chl*	mg m^−3^	O’Reilly et al., 1998; Hu et al., 2012b
Chlorophyll-a derived from band ratio	–	*Chl*_*BR*_	mg m^−3^	O’Reilly et al., 1998
Chlorophyll-a derived from line height	–	*Chl*_*LH*_	mg m^−3^	Hu et al., 2012b
Diffuse attenuation coefficient at 490 nm	OC	*K*_*d*,490_	m^−1^	Mueller, 2000
Particulate organic carbon	OC	POC	mg m^−3^	Stramski et al., 2008a
Normalized fluorescent line height	OC	nflh	mW cm^−2^ µm^−1^ sr^−1^	Behrenfeld et al., 2009
Absorption coefficient of total non-water components 443 nm	IOP	*a*_*nw*,*443*_	m^−1^	Werdell et al., 2013
Absorption coefficient of phytoplankton at 443 nm	IOP	*a*_*ϕ*,*443*_	m^−1^	Werdell et al., 2013
Absorption coefficient of colored dissolved and detrital matter at 443 nm	IOP	*a*_*dg*,*443*_	m^−1^	Werdell et al., 2013
Particulate backscattering coefficient at 443 nm	IOP	*b*_*bp*,*443*_	m^−1^	Werdell et al., 2013

* Note that NASA’s standard Chl product is a dynamic blend of Chl_BR_ and Chl_LH_.

**TABLE 2 |:** Log-normal statistics comparing Monte Carlo (MC) and first-order first-moment (FOFM) uncertainty calculations for *R*_*rs*_ with spectrally flat, uncorrelated 5% relative uncertainty.

	Derived product uncertainty	
Product	Bias	Slope
*Chl* (all)	0.95	0.96
*Chl*_*BR*_	1.00	1.00
*Chl*_*LH*_	0.99	1.00
*Chl*_blended_*	0.73	0.72
*K*_*d*,490_	0.99	1.00
*POC*	0.99	1.00
*nflh*	0.99	1.00
*a*_*nw*,*443*_	0.99	1.00
*a*_*ϕ*,*443*_	0.98	1.00
*a*_*dg*,*443*_	0.98	1.00
*b*_*bp*,*443*_	0.99	0.98

* Blended LH and BR Chl product span 0.134–0.165 mg m^-3^.

**TABLE 3 |:** OC products and associated uncertainties derived via MC method with 5%, uncorrelated relative uncertainty in *R*_*rs*_.

	Derived value		Absolute uncertainty		Relative uncertainty (%)	
Product	Range	Median	Range	Median	Range	Median
*Chl* (mg m^−3^)	3.96 × 10^−2^−1.27	0.110	2.56 × 10^−5^-0.231	7.00 × 10^−3^	1.73–18.2	9.74
*K*_*d,490*_ (m^−1^)	2.01 × 10^−2^−0.131	2.91 ×10^−2^	1.19 × 10^−3^−1.36 × 10^−2^	2.68 × 10^−3^	5.92–10.5	8.94
*POC* (mg m^−3^)	18.8–203.4	33.1	1.37–14.6	2.44	7.11–7.60	7.37
*nflh* (mW cm^−2^ µm^−1^ sr^−1^)	5.25 × 10^−6^−2.74 × 10^−2^	2.20 × 10^−3^	3.18 × 10^−4^-4.47 × 10^−3^	9.86 × 10^−4^	14.8–1.7 × 10^4^	41.9

**TABLE 4 |:** OC products and associated uncertainties derived via FOFM method with 5%, uncorrelated relative uncertainty in *R*_*rs*_.

	Derived value		Absolute uncertainty		Relative uncertainty (%)	
Product	Range	Median	Range	Median	Range	Median
*Chl* (mg m^−3^)	3.96 × 10^−2^−1.28	0.110	3.89 × 10^−5^-0.230	6.70 × 10^−3^	0.26–18.7	9.67
*K*_*d,490*_ (m^−1^)	2.01 × 10^−2^−0.131	2.91 × 10^−2^	1.18 × 10^−3^−1.33 × 10^−2^	2.68 × 10^−3^	5.86–10.2	8.91
*POC* (mg m^−3^)	18.8–203.4	33.1	1.37–14.9	2.42	7.31	
*nflh* (mW cm^−2^ µm^−1^ sr^−1^)	2.05 × 10^−6^−2.73 × 10^−2^	2.19E × 10^−3^	3.21 × 10^−4^−4.43 × 10^−3^	9.87 × 10^−4^	15.1–3.24 × 10^4^	42.1

* Relative uncertainties in POC computed using FOFM method were constant over the dynamic range.

**TABLE 5 |:** IOP products and associated uncertainties derived using MC method with 5%, uncorrelated relative uncertainty in *R*_*rs*_.

	Derived value		Absolute uncertainty		Relative uncertainty (%)	
Product	Range	Median	Range	Median	Range	Median
*a*_*nw*_(443) (m^−1^)	9.40 × 10^−3^−0.127	0.0185	1.79 × 10^−3^−1.13×10^−2^	2.31 × 10^−3^	8.16–19.4	12.6
*a*_*ϕ*_(443) (m^−1^)	5.80 × 10^−3^−9.43 ×10^−2^	9.60 × 10^−3^	1.63 × 10^−3^−9.68 × 10^−3^	2.04 × 10^−3^	10.0–29.2	21.4
*a*_*dg*_(443) (m^−1^)	3.50 × 10^−3^−3.72 ×10^−2^	8.71 × 10^−3^	6.66 × 10^−4^−5.90 × 10^−3^	1.07 × 10^−3^	7.92–19.9	14.5
*b*_*bp*_(443) (m^−1^)	4.18 × 10^−4^−4.00 × 10^−3^	1.08 × 10^−3^	8.98 × 10^−5^−2.25E × 10^−4^	1.34 × 10^−4^	5.57–34.1	13.8

**TABLE 6 |:** IOP products and associated uncertainties derived using FOFM method with 5%, uncorrelated relative uncertainty in *R*_*rs*_.

	Derived value		Absolute uncertainty		Relative uncertainty (%)	
Product	Range	Median	Range	Median	Range	Median
*a*_*nw,443*_ (m^−1^)	9.42 × 10^−3^−0.127	0.0185	1.79 × 10^−3^-1.03 × 10^−2^	2.26 × 10^−3^	8.12–19.1	12.2
*a*_*ϕ*,*443*_ (m^−1^)	5.86 × 10^−3^−9.45 × 10^−2^	9.63E-3	1.64 × 10^−4^−8.73 × 10^−3^	2.00 × 10^−3^	9.02–28.6	20.8
*a*_*dg,443*_ (m^−1^)	3.51 × 10^−3^−3.70 × 10^−2^	8.73E-3	6.51 × 10^−4^−5.63 × 10^−3^	1.05 × 10^−3^	7.93–18.9	14.1
*b*_*bp,443*_ (m^−1^)	4.16 × 10^−4^−4.01×10^−3^	1.00E-3	9.00 × 10^−5^−2.11 × 10^−4^	1.33 × 10^−4^	5.25–34.1	13.9

**TABLE 7 |:** GIOP model-misfit uncertainties estimated using the evaluation *R*_*rs*_ dataset.

	Absolute uncertainty (m−^1^)		Relative uncertainty (%)		Difference between absolute data and absolute model misfit uncertainties* (%)
Product	Range	Median	Range	Median	Median
*a*_*tw*,*443*_ (m^−1^)	3.88 × 10^−4^ −5.71 × 10^−3^	4.87 × 10^−4^	1.26–5.70	3.15	−77
*a*_*ϕ*,*443*_ (m^−1^)	3.67 × 10^−4^−5.25 × 10^−3^	4.54 × 10^−4^	3.02–9.09	4.68	−77
*a*_*dg*,*443*_ (m^−1^)	1.07 × 10^−4^−2.26 × 10^−3^	1.434 × 10^−4^	0.81–7.48	2.86	−86
*b*_*bp*,*443*_ (m^−1^)	2.94 × 10^−5^−2.17 × 10^−4^	5.22 × 10^−5^	1.57–9.58	4.52	−61

* Differences between median absolute model uncertainties in this table and median absolute radiometric (data) uncertainties (column RU: Hu in Table 9).

**TABLE 8 |:** Median OC data product uncertainties computed as relative uncertainties (RU) in *R*_*rs*_ vary.

	Median absolute uncertainties				Median relative uncertainties (%)			
	RU: 1%	RU: 5%	RU: 10%	RU: Hu	RU: 1%	RU: 5%	RU: 10%	RU: Hu
**Product**								
*Chl* (mg m^−3^)	1.52 × 10^−3^	6.70 × 10^−3^	1.46 × 10^−2^	6.50 × 10^−3^	1.96	9.67	19.35	8.29
*K*_*d,490*_(m^−1^)	5.37 × 10^−4^	2.68 × 10^−3^	5.36 × 10^−3^	5.07 × 10^−3^	1.78	8.91	17.8	17.3
*POC* (mg m^−3^)	4.84 × 10^−1^	2.42	4.84	4.38	1.46	7.31	14.6	13.1
*nflh* (mW cm^−2^ µm^−1^ sr^−1^)	1.97 × 10^−4^	9.87 × 10^−4^	1.97 × 10^−3^	4.47 × 10^−3^	8.41	42.1	84.1	197.6

**TABLE 9 |:** Median IOP data product uncertainties computed as relative uncertainties (RU) in *R*_*rs*_ vary.

	Median absolute uncertainties				Median relative uncertainties (%)			
	RU: 1%	RU: 5%	RU: 10%	RU: Hu	RU: 1%	RU: 5%	RU: 10%	RU: Hu
**Product**								
*a*_*tw*,*443*_ (m^−1^)	4.52 × 10^−4^	2.26 × 10^−3^	4.52 × 10^−3^	2.76 × 10^−3^	2.45	12.2	24.5	15.1
*a*_*ϕ*,*443*_ (m^−1^)	4.00 × 10^−4^	2.00 × 10^−3^	4.00 × 10^−3^	2.42 × 10^−3^	4.15	20.8	41.6	23.8
*a*_*dg*,*443*_ (m^−1^)	2.11 × 10^−4^	1.05 × 10^−3^	2.11 × 10^−3^	1.33 × 10^−3^	2.82	14.1	28.2	15.9
*b*_*bp*,*443*_ (m^−1^)	2.67 × 10^−5^	1.33 × 10^−4^	2.67 × 10^−4^	1.73 × 10^−4^	2.78	13.9	27.9	17.9

**TABLE 10 |:** Simplified random uncertainty budgets for two POC models.

Algorithm	Median derived value (mg m^−3^)	Median absolute uncertainty in mg m^−3^ (median relative uncertainty in %)		
		Data	Model	Measurement
Stramski et al., 2008a	33.1	4.40	0.94	4.50 (16.6)
Rasse et al., 2017	37.8	6.96	17.30	18.6 (49.2)

Median absolute uncertainties and median relative uncertainties were computed using our R_rs_ evaluation dataset with Hu spectrally-dependent, uncorrelated radiometric uncertainties and basic knowledge of model coefficient uncertainty. We note that these data are intended to illustrate how one might formulate measurement uncertainty budgets. These data are not intended for algorithm comparison purposes.

## Footnotes

1 NASA Goddard Space Flight Center, Ocean Ecology Laboratory, & Ocean Biology Processing Group. Sea-viewing Wide Field-of-view Sensor (SeaWiFS) L1 Data (data/10.5067/ORBVIEW-2/SEAWIFS/L1/DATA/1).

## References

[R1] Angal A, Xiong X, Sun J, and Geng X (2015). On-orbit noise characterization of MODIS reflective solar bands. J. Appl. Remote Sens 9:094092.

[R2] Antoine D, d’Ortenzio F, Hooker SB, Bécu G, Gentili B, Tailliez D, (2008). Assessment of uncertainty in the ocean reflectance determined by three satellite ocean color sensors (MERIS, SeaWiFS and MODIS-A) at an offshore site in the Mediterranean Sea (BOUSSOLE project). J. Geophys. Res. Oceans 113, 1–22. doi: 10.1029/2007JC004472

[R3] Bailey SW, Franz BA, and Werdell PJ (2010). Estimation of near-infrared water-leaving reflectance for satellite ocean color data processing. Opt. Express 18, 7521–7527. doi: 10.1364/OE.18.007521 20389774

[R4] Bailey SW, and Werdell PJ (2006). A multi-sensor approach for the on-orbit validation of ocean color satellite data products. Remote Sens. Environ 102, 12–23. doi: 10.1016/j.rse.2006.01.015

[R5] Balch WM, Gordon HR, Bowler BC, Drapeau DT, and Booth ES (2005). Calcium carbonate measurements in the surface global ocean based on Moderate-Resolution Imaging Spectroradiometer data. J. Geophys. Res. Oceans 110, 1–21. doi: 10.1029/2004JC002560

[R6] Behrenfeld MJ, Westberry TK, Boss ES, O’Malley RT, Siegel DA, Wiggert JD, (2009). Satellite-detected fluorescence reveals global physiology of ocean phytoplankton. Biogeosciences 6, 779–794. doi: 10.5194/bg-6-779-2009

[R7] Campbell JW (1995). The lognormal distribution as a model for bio-optical variability in the sea. J. Geophys. Res. Oceans 100, 13237–13254. doi: 10.1029/95JC00458

[R8] Chase AP, Boss E, Cetinić I, and Slade W (2017). Estimation of phytoplankton accessory pigments from hyperspectral reflectance spectra: toward a global algorithm. J. Geophys. Res. Oceans 122, 9725–9743. doi: 10.1002/2017JC012859

[R9] Eplee JRE, Patt FS, Barnes RA, and McClain CR (2007). SeaWiFS long-term solar diffuser reflectance and sensor noise analyses. Appl. Opt 46, 762–773. doi: 10.1364/AO.46.000762 17279164

[R10] Franz BA, Behrenfeld MJ, Siegel DA, and Signorini SR (2017). Global ocean phytoplankton [in: State of the Climate in 2016]. Bull. Amer. Meteor. Soc 99, S94–S96. doi: 10.1175/2018BAMSStateoftheClimate.1

[R11] Gillis DB, Bowles JH, Montes MJ, and Moses WJ (2018). Propagation of sensor noise in oceanic hyperspectral remote sensing. Opt. Express 26, A818–A831. doi: 10.1364/OE.26.00A818 30184914

[R12] Gordon HR, Brown OB, Evans RH, Brown JW, Smith RC, Baker KS, (1988). A semianalytic radiance model of ocean color. J. Geophys. Res. Atmos 93, 10909–10924. doi: 10.1029/JD093iD09p10909

[R13] Gordon HR, and Wang M (1994). Retrieval of water-leaving radiance and aerosol optical thickness over the oceans with SeaWiFS: a preliminary algorithm. Appl. Opt 33, 443–452. doi: 10.1364/AO.33.000443 20862036

[R14] Gould WG, McCarthy SE, Coelho E, Shulman I, and Richman JG (2014). Combining satellite ocean color and hydrodynamic model uncertainties in bio-optical forecasts. J. Appl. Remote Sens 8:083652 doi: 10.1117/1.JRS.8.083652

[R15] Hooker SB, Esaias WE, Feldman GC, Gregg WW, and McClain CR (1992). An Overview of SeaWiFS and Ocean-Color, NASA Tech. Memo. 104566, Vol. 1, eds Hooker SB, and Firestone ER Greenbelt, MD: NASA Goddard Space Flight Center, 24.

[R16] Hooker SB, and McClain CR (2000). The calibration and validation of SeaWiFS data. Prog. Oceanogr 45, 427–465. doi: 10.1016/S0079-6611(00)00012-4

[R17] Hu C, Feng L, and Lee Z (2013). Uncertainties of SeaWiFS and MODIS remote sensing reflectance: implications from clear water measurements. Remote Sens. Environ 133, 168–182. doi: 10.1016/j.rse.2013.02.012

[R18] Hu C, Feng L, Lee Z, Davis CO, Mannino A, McClain CR, (2012a). Dynamic range and sensitivity requirements of satellite ocean color sensors: learning from the past. Appl. Opt 51, 6045–6062. doi: 10.1364/AO.51.006045 22945151

[R19] Hu C, Lee Z, and Franz B (2012b). Chlorophyll a algorithms for oligotrophic oceans: a novel approach based on three-band reflectance difference. J. Geophys. Res. Oceans 117(C1), 1–25. doi: 10.1029/2011JC007395

[R20] IOCCG (2008). Why Ocean Colour? The Societal Benefits of Ocean- Colour Technology, Vol. 7 Dartmouthn, NS: IOCCG.

[R21] Jay S, Guillaume M, Chami M, Minghelli A, Deville Y, Lafrance B, (2018). Predicting minimum uncertainties in the inversion of ocean color geophysical parameters based on Cramer-Rao bounds. Opt. Express 26, A1–A18. doi: 10.1364/OE.26.0000A1 29402051

[R22] JCGM (2008). Evaluation of Measurement Data - Guide to the Expression of Uncertainty in Measurement JCGM 100:2008.

[R23] Lamquin N, Mangin A, Mazeran C, Bourg B, Bruniquel V, and D’Andon OF (2013). OLCI L2 Pixel-by-Pixel Uncertainty Propagation in OLCI Clean Water Branch. ESA ATBD ref. S3-L2-SD-01-C01-ACR-TN

[R24] Lee Z, Arnone R, Hu C, Werdell PJ, and Lubac B (2010). Uncertainties of optical parameters and their propagations in an analytical ocean color inversion algorithm. Appl. Opt 49, 369–381. doi: 10.1364/AO.49.000369 20090801

[R25] Lee Z, Carder KL, and Arnone RA (2002). Deriving inherent optical properties from water color: a multiband quasi-analytical algorithm for optically deep waters. Appl. Opt 41, 5755–5772. doi: 10.1364/AO.41.005755 12269575

[R26] Lee Z, Du K, Voss KJ, Zibordi G, Lubac B, Arnone R, (2011). An inherent-optical-property-centered approach to correct the angular effects in water-leaving radiance. Appl. Opt 50, 3155–3167. doi: 10.1364/AO.50.003155 21743515

[R27] Maritorena S, d’Andon OHF, Mangin A, and Siegel DA (2010). Merged satellite ocean color data products using a bio-optical model: characteristics, benefits and issues. Remote Sens. Environ 114, 1791–1804. doi: 10.1016/j.rse.2010.04.002

[R28] McClain CR (2009). A decade of satellite ocean color observations. Ann. Rev. Mar. Sci 1, 19–42. doi: 10.1146/annurev.marine.010908.163650 21141028

[R29] McClain CR, Feldman GC, and Hooker SB (2004). An overview of the SeaWiFS project and strategies for producing a climate research quality global ocean bio-optical time series. Deep Sea Res. Part II Topical Stud. Oceanogr 51, 5–42. doi: 10.1016/j.dsr2.2003.11.001

[R30] McKinna LIW, Werdell PJ, and Proctor CW (2016). Implementation of an analytical Raman scattering correction for satellite ocean-color processing. Opt. Express 24, A1123–A1137. doi: 10.1364/OE.24.0A1123 27410899

[R31] Mekid S, and Vaja D (2008). Propagation of uncertainty: expressions of second and third order uncertainty with third and fourth moments. Measurement 41, 600–609. doi: 10.1016/j.measurement.2007.07.004

[R32] Melin F (2010). Global distribution of the random uncertainty associated with satellite-derived Chl a. IEEE Geosci. Remote Sens. Lett 7, 220–224. doi: 10.1109/LGRS.2009.2031825

[R33] Mélin F, Sclep G, Jackson T, and Sathyendranath S (2016). Uncertainty estimates of remote sensing reflectance derived from comparison of ocean color satellite data sets. Remote Sens. Environ 177, 107–124. doi: 10.1016/j.rse.2016.02.014

[R34] Moore TS, Campbell JW, and Dowell MD (2009). A class-based approach to characterizing and mapping the uncertainty of the MODIS ocean chlorophyll product. Remote Sens. Environ 113, 2424–2430. doi: 10.1016/j.rse.2009.07.016

[R35] Morel A, Antoine D, and Gentili B (2002). Bidirectional reflectance of oceanic waters: accounting for Raman emission and varying particle scattering phase function. Appl. Opt 41, 6289–6306. doi: 10.1364/AO.41.006289 12396179

[R36] Mueller JL (2000). “SeaWiFS algorithm for the diffuse attenuation coefficient, K(490), using water-leaving radiances at 490 and 555 nm,” in eds Hooker SB and Firestone E, R NASA Technical Memorandum 2000–206829, Vol. 11 (Greenbelt, MD: NASA Goddard Space Flight Center), 51.

[R37] Neukermans G, Ruddick K, Bernard E, Ramon D, Nechad B, and Deschamps P-Y (2009). Mapping total suspended matter from geostationary satellites: a feasibility study with SEVIRI in the Southern North Sea. Opt. Express 17, 14029–14052. doi: 10.1364/OE.17.014029 19654812

[R38] Novak MG, Cetinić I, Chaves JE, and Mannino A (2018). The adsorption of dissolved organic carbon onto glass fiber filters and its effect on the measurement of particulate organic carbon: a laboratory and modeling exercise. Limnol. Oceanogr. Methods 16, 356–366. doi: 10.1002/lom3.10248 30271309 PMC6155487

[R39] O’Reilly JE, Maritorena S, Mitchell BG, Siegel DA, Carder KL, Garver SA, (1998). Ocean color chlorophyll algorithms for SeaWiFS. J. Geophys. Res. Oceans 103, 24937–24953. doi: 10.1029/98JC02160

[R40] PACE Science Definition Team (2018). Pre-Aerosol, Clouds, and ocean Ecosystem (PACE) Mission Science Definition Team Report Greenbelt, MD.

[R41] Putko MM, Taylor IIIAC, Newman PA, and Green LL (2001). Approach for input uncertainty propagation and robust design in CFD using sensitivity derivatives. J. Fluids Eng 124, 60–69. doi: 10.1115/1.1446068

[R42] Qi L, Lee Z, Hu C, and Wang M (2017). Requirement of minimal signal-to-noise ratios of ocean color sensors and uncertainties of ocean color products. J. Geophys. Res. Oceans 122, 2595–2611. doi: 10.1002/2016JC012558

[R43] Rasse R, Dall’Olmo G, Graff J, Westberry TK, van Dongen-Vogels V, and Behrenfeld MJ (2017). Evaluating optical proxies of particulate organic carbon across the surface atlantic ocean. Front. Marine Sci 4, 1–18. doi: 10.3389/fmars.2017.00367

[R44] Refsgaard JC, van der Sluijs JP, Højberg AL, and Vanrolleghem PA (2007). Uncertainty in the environmental modelling process – a framework and guidance. Environ. Model. Softw 22, 1543–1556. doi: 10.1016/j.envsoft.2007.02.004

[R45] Salama MS, Dekker A, Su Z, Mannaerts CM, and Verhoef W (2009). Deriving inherent optical properties and associated inversion-uncertainties in the Dutch Lakes. Hydrol. Earth Syst. Sci 13, 1113–1121. doi: 10.5194/hess-13-1113-2009

[R46] Salama MS, Mélin F, and Van der Velde R (2011). Ensemble uncertainty of inherent optical properties. Opt. Express 19, 16772–16783. doi: 10.1364/OE.19.016772 21935039

[R47] Seegers BN, Stumpf RP, Schaeffer BA, Loftin KA, and Werdell PJ (2018). Performance metrics for the assessment of satellite data products: an ocean color case study. Opt. Express 26, 7404–7422. doi: 10.1364/OE.26.007404 29609296 PMC5894891

[R48] Stramski D, Reynolds RA, Babin M, Kaczmarek S, Lewis MR, Rottgers R, (2008a). Relationships between the surface concentration of particulate organic carbon and optical properties in the eastern South Pacific and eastern Atlantic Oceans. Biogeosciences 5, 171–201. doi: 10.5194/bg-5-171-2008

[R49] Stramski D, Reynolds RA, Babin M, Kaczmarek S, Lewis MR, Röttgers R, (2008b). Concentration of particulate organic carbon and optical properties in the eastern South Pacific and eastern Atlantic Oceans. Supplement to: Stramski, D et al. (2008): Relationships between the surface concentration of particulate organic carbon and optical properties in the eastern South Pacific and eastern Atlantic Oceans. Biogeosciences 5, 171–201. doi: 10.5194/bg-5-171-2008

[R50] Wang P, Boss ES, and Roesler C (2005). Uncertainties of inherent optical properties obtained from semianalytical inversions of ocean color. Appl. Opt 44, 4074–4085. doi: 10.1364/AO.44.004074 16004055

[R51] Werdell PJ, and Bailey SW (2005). An improved *in-situ* bio-optical data set for ocean color algorithm development and satellite data product validation. Remote Sens. Environ 98, 122–140. doi: 10.1016/j.rse.2005.07.001

[R52] Werdell PJ, Franz BA, Bailey SW, Feldman GC, Boss E, Brando VE, (2013). Generalized ocean color inversion model for retrieving marine inherent optical properties. Appl. Opt 52, 2019–2037. doi: 10.1364/AO.52.002019 23545956

[R53] Westberry TK, Boss E, and Lee Z (2013). Influence of Raman scattering on ocean color inversion models. Appl. Opt 52, 5552–5561. doi: 10.1364/AO.52.005552 23913078

