# Supplementary material for: Approach for Propagating Radiometric Data Uncertainties Through NASA Ocean Color Algorithms
Source: Front Earth Sci (Lausanne). Author manuscript; Available in PMC 2020 Jul 18. (PMC7344266; doi:10.3389/feart.2019.00176)
Supplement: Appendices A-F [file NIHMS1538702-supplement-Appendices_A-F.pdf]

Approach for propagating radiometric data uncertainties through NASA ocean color algorithms: Appendices A-F

Lachlan I.W. McKinna[1,4*], Ivona Cetinić[2,4], Alison P. Chase[3], P. Jeremy Werdell[4]

[1]Go2Q Pty Ltd, Sunshine Coast, QLD, Australia

[2]GESTAR/Universities Space Research Association, Columbia, MD, USA

[3]School of Marine Sciences, University of Maine, Orono, ME, USA

[4]Ocean Ecology Laboratory, NASA Goddard Spaceflight Center, Code 616, Greenbelt, MD

*** Correspondence:**
Lachlan I.W. McKinna
lachlan.mckinna@go2q.com.au

Tables of Contents

Appendix A: Chlorophyll concentration and uncertainty 2
Appendix B: Diffuse attenuation coefficient and uncertainty 7
Appendix C: Particulate organic carbon and uncertainty 9
Appendix D: Normalized fluorescent line height and uncertainty 11
Appendix E: Inherent optical properties and uncertainty 13
Appendix F: Approximating correlated uncertainties due to sensor noise 20

Appendix A: Chlorophyll concentration and uncertainty

NASA's standard chlorophyll-a pigment (*Chl*; mg m^{-3}) algorithm is a combination of a blue-to-green maximum band ratio algorithm (Chl_{BR}) (O'Reilly et al., 1998) and a chlorophyll index (line height) algorithm (Chl_{LH}) (Hu et al., 2012). During pixel-by-pixel processing, both Chl_{BR} and Chl_{LH} are computed.

A.1 Band ratio Chl model

Chl_{BR} is returned as the solution when Chl_{BR} > 0.2 mg m^{-3} and is computed as follows:

$$Chl_{BR} = 10^{\alpha} \qquad [A1]$$

which has the derivative:

$$\frac{\partial Chl_{BR}}{\partial \alpha} = \log(10)10^{\alpha} \qquad [A2]$$

where α is a polynomial function. The order of the polynomial, *N=4*, and the coefficients a_i are sensor dependent. Specifically, α is expressed as:

$$\alpha = \sum_{i=1}^{4} a_i \left[LR\right]^i \qquad [A3]$$

and has the derivative (assuming the coefficients, a_i, have no uncertainties):

$$\frac{\partial \alpha}{\partial LR} = \sum_{i=0}^{4} i a_i \left[LR\right]^{i-1} . \qquad [A4]$$

The log-ratio, *LR*, term is:

$$LR = \log_{10}\left(\frac{R_{rs,b}}{R_{rs,g}}\right) \qquad [A5]$$

where $R_{rs,b}$ and $R_{rs,g}$ are remote sensing reflectances centered on blue and green sensor bands, respectively.

The partial derivatives of Eq. A5 are:

$$\frac{\partial LR}{\partial R_{rs,b}} = \frac{1}{\log(10)} \frac{1}{R_{rs,g}} , \qquad [A6a]$$

and

$$\frac{\partial LR}{\partial R_{rs,g}} = -\frac{1}{\log(10)} \frac{R_{rs,b}}{R_{rs,g}^2} . \qquad [A6b]$$

In this analysis, we consider the Chl_{BR} formulation known as "OC4" tuned for the SeaWiFS sensor where the green reference band, λ_g, is centered on 555 nm and the blue band is selected as follows:

$$R_{rs,b} = \max\{R_{rs,443}, R_{rs,490}, R_{rs,510}\} \qquad [A7]$$

The fourth order polynomial coefficients a_i were determined empirically from a comprehensive in situ data set of coincident *Chl* and $R_{rs}(\lambda)$ data *(Werdell & Bailey, 2005)*. For OC4, the coefficients a_0, a_1, a_2, a_3, a_4, have values of 0.3272, -2.9940, 2.7218, -1.2259, and -0.5683, respectively.

The variance in Chl_{BR} is thus estimated as:

$$u^2(Chl_{BR}) = \left(\frac{\partial Chl_{BR}}{\partial R_{rs,b}}\right)^2 u^2(R_{rs,b}) + \left(\frac{\partial Chl_{BR}}{\partial R_{rs,555}}\right)^2 u^2(R_{rs,g}) + 2\frac{\partial Chl_{BR}}{\partial R_{rs,b}}\frac{\partial Chl_{BR}}{\partial R_{rs,555}} u(R_{rs,b}, R_{rs,555})$$

[A8]

where $u^2(R_{rs,b})$ and $u^2(R_{rs,555})$ are the variances of $R_{rs,b}$ and $R_{rs,555}$, respectively and the $u(R_{rs,b},R_{rs,555})$ is the error covariance of $R_{rs,b}$ and $R_{rs,555}$. The partial derivatives in Eq. A8 are computed as:

$$\frac{\partial Chl_{BR}}{\partial R_{rs,b}} = \frac{\partial Chl_{BR}}{\partial \alpha}\frac{\partial \alpha}{\partial LR}\frac{\partial LR}{\partial R_{rs,b}} \qquad [A9a]$$

and

$$\frac{\partial Chl_{BR}}{\partial R_{rs,555}} = \frac{\partial Chl_{BR}}{\partial \alpha}\frac{\partial \alpha}{\partial LR}\frac{\partial LR}{\partial R_{rs,555}} \ . \qquad [A9b]$$

A.2 Line height Chl model

Chl_{LH} is returned as the solution when $Chl_{LH} \leq 0.15$ mg m^{-3} and is computed as follows:

$$Chl_{LH} = 10^{\beta}, \qquad [A10]$$

which has the derivative:

$$\frac{\partial Chl_{LH}}{\partial \beta} = \log(10)10^{\beta}, \qquad [A10]$$

where,

$$\beta = -0.4909 + 191.5690 LH \qquad [A11]$$

which has the derivative:

$$\frac{\partial \beta}{\partial LH} = 191.6590 \ . \qquad [A13]$$

The *LH* term has the form:

$$LH = R_{rs,555} - \left[R_{rs,443} + \frac{(555-443)}{(670-443)} \left(R_{rs,670} - R_{rs,443} \right) \right]. \qquad [A14]$$

with the following partial derivatives:

$$\frac{\partial LH}{\partial R_{rs,443}} = \frac{(555-443)}{(670-443)} - 1 \,, \qquad [A15a]$$

$$\frac{\partial LH}{\partial R_{rs,555}} = 1, \qquad [A15b]$$

and

$$\frac{\partial LH}{\partial R_{rs,670}} = -\frac{(555-443)}{(670-443)} \,. \qquad [A15c]$$

The variance in Chl_{LH} can then be estimated as:

$$\begin{aligned} u^2(Chl_{LH}) = & \left(\frac{\partial Chl_{LH}}{\partial R_{rs,443}} \right)^2 u^2(R_{rs,443}) + \left(\frac{\partial Chl_{LH}}{\partial R_{rs,555}} \right)^2 u^2(R_{rs,555}) + \left(\frac{\partial Chl_{LH}}{\partial R_{rs,670}} \right)^2 u^2(R_{rs,670}) \ldots \\ & + 2 \frac{\partial Chl_{LH}}{\partial R_{rs,443}} \frac{\partial Chl_{LH}}{\partial R_{rs,555}} u(R_{rs,443}, R_{rs,555}) + 2 \frac{\partial Chl_{LH}}{\partial R_{rs,443}} \frac{\partial Chl_{LH}}{\partial R_{rs,670}} u(R_{rs,443}, R_{rs,670}) \ldots \qquad [A16] \\ & + 2 \frac{\partial Chl_{LH}}{\partial R_{rs,555}} \frac{\partial Chl_{LH}}{\partial R_{rs,670}} u(R_{rs555}, R_{rs,670}) \end{aligned}$$

where $u^2(R_{rs,443})$, $u^2(R_{rs,555})$ and $u^2(R_{rs,670})$ are the variances of $R_{rs,443}$, $R_{rs,555}$, and $R_{rs,670}$, respectively. The term $u(R_{rs,443}, R_{rs,555})$ is the error covariance of $R_{rs,443}$ and $R_{rs,555}$, $u(R_{rs,443}, R_{rs,670})$ is the error covariance of $R_{rs,443}$ and $R_{rs,670,}$ and $(R_{rs,555}, R_{rs,670})$ is the error covariance of $R_{rs,555}$, and $R_{rs,670}$. The partial derivatives in Eq. A16 are computed as:

$$\frac{\partial Chl_{LH}}{\partial R_{rs,443}} = \frac{\partial Chl_{LH}}{\partial \beta} \frac{\partial \beta}{\partial LH} \frac{\partial LH}{\partial R_{rs,443}} \,, \qquad [A17a]$$

$$\frac{\partial Chl_{LH}}{\partial R_{rs,555}} = \frac{\partial Chl_{LH}}{\partial \beta} \frac{\partial \beta}{\partial LH} \frac{\partial LH}{\partial R_{rs,555}} \,, \qquad [A17b]$$

and

$$\frac{\partial Chl_{LH}}{\partial R_{rs,670}} = \frac{\partial Chl_{LH}}{\partial \beta} \frac{\partial \beta}{\partial LH} \frac{\partial LH}{\partial R_{rs,670}} . \qquad [A17c]$$

A.3 Blended Chl product

For intermediate conditions where $Chl_{LH} > 0.15$ mg m^{-3} and $Chl_{BR} \leq 0.2$ mg m^{-3}, Chl_{LH} and Chl_{BR} values are blended together and returned as the solution (Hu et al., 2012). The blending is performed as

follows:

$$Chl_{blend} = Chl_{LH}\frac{(0.20 - Chl_{LH})}{0.2 - 0.15} + Chl_{BR}\frac{(Chl_{LH} - 0.15)}{0.2 - 0.15} \; . \qquad \text{[A18]}$$

The variance in Chl_{blend} is estimated as follows:

$$u^2(Chl_{blend}) = \left(\frac{\partial Chl_{blend}}{\partial R_{rs,443}}\right)^2 u^2(R_{rs,443}) + \left(\frac{\partial Chl_{blend}}{\partial R_{rs,490}}\right)^2 u^2(R_{rs,490}) + \left(\frac{\partial Chl_{blend}}{\partial R_{rs,510}}\right)^2 u^2(R_{rs,510})\ldots$$
$$+\left(\frac{\partial Chl_{blend}}{\partial R_{rs,555}}\right)^2 u^2(R_{rs,555}) + \left(\frac{\partial Chl_{blend}}{\partial R_{rs,670}}\right)^2 u^2(R_{rs,670})\ldots$$
$$+2\frac{\partial Chl_{blend}}{\partial R_{rs,443}}\frac{\partial Chl_{blend}}{\partial R_{rs,490}}u(R_{rr,443}, R_{rs,490}) + 2\frac{\partial Chl_{blend}}{\partial R_{rs,443}}\frac{\partial Chl_{blend}}{\partial R_{rs,510}}u(R_{rs,443}, R_{rs,510})\ldots$$
$$+2\frac{\partial Chl_{blend}}{\partial R_{rs,443}}\frac{\partial Chl_{blend}}{\partial R_{rs,555}}u(R_{rs,443}, R_{rs,555}) + 2\frac{\partial Chl_{blend}}{\partial R_{rs,443}}\frac{\partial Chl_{blend}}{\partial R_{rs670}}u(R_{rs,443}, R_{rs,670})\ldots$$
$$+2\frac{\partial Chl_{blend}}{\partial R_{rs,490}}\frac{\partial Chl_{blend}}{\partial R_{rs,510}}u(R_{rs,490}, R_{rs,510}) + 2\frac{\partial Chl_{blend}}{\partial R_{rs,490}}\frac{\partial Chl_{blend}}{\partial R_{rs,555}}u(R_{rs,490}, R_{rs,555})\ldots$$
$$+2\frac{\partial Chl_{blend}}{\partial R_{rs,490}}\frac{\partial Chl_{blend}}{\partial R_{rs,670}}u(R_{rs,490}, R_{rs,670}) + 2\frac{\partial Chl_{blend}}{\partial R_{rs,510}}\frac{\partial Chl_{blend}}{\partial R_{rs,555}}u(R_{rs,510}, R_{rs,555})\ldots$$
$$+2\frac{\partial Chl_{blend}}{\partial R_{rs,510}}\frac{\partial Chl_{blend}}{\partial R_{rs,670}}u(R_{rs,510}, R_{rs,670}) + 2\frac{\partial Chl_{blend}}{\partial R_{rs,555}}\frac{\partial Chl_{blend}}{\partial R_{rs670}}u(R_{rs,555}, R_{rs,670}).$$

[A19]

Where $u^2(R_{rs,i})$ terms are variances and $u(R_{rs,i}, R_{rs,j})$ terms are error covariances. The partial derivatives in Equation A19 are:

$$\frac{\partial Chl_{blend}}{\partial R_{rs,i}} = \frac{1}{0.2 - 0.15}\left[0.2\frac{\partial Chl_{LH}}{\partial R_{rs,i}} - \frac{\partial Chl_{LH}^2}{\partial R_{rs,i}} + \frac{\partial(Chl_{BR}Chl_{LH})}{\partial R_{rs,i}} - 0.15\frac{\partial Chl_{BR}}{\partial R_{rs,i}}\right].$$

[A20]

and

$$\frac{\partial Chl_{LH}^2}{\partial R_{rs,i}} = 2Chl_{LH}\frac{\partial Chl_{LH}}{\partial R_{rs,i}}, \qquad \text{[A21a]}$$

$$\frac{\partial(Chl_{LH}Chl_{BR})}{\partial R_{rs,i}} = Chl_{LH}\frac{\partial Chl_{BR}}{\partial R_{rs,i}} + Chl_{BR}\frac{\partial Chl_{LH}}{\partial R_{rs,i}}. \qquad \text{[A21b]}$$

$$\frac{\partial Chl_{LH}Chl_{BR}}{\partial R_{rs,i}} = Chl_{LH}\frac{\partial Chl_{BR}}{\partial R_{rs,i}} + Chl_{BR}\frac{\partial Chl_{LH}}{\partial R_{rs,i}}$$

References

Hu, C., Lee, Z., & Franz, B. (2012). Chlorophyll a algorithms for oligotrophic oceans: A novel approach based on three-band reflectance difference. *Journal of Geophysical Research: Oceans, 117*(C1), n/a-n/a. doi:10.1029/2011JC007395

O'Reilly, J. E., Maritorena, S., Mitchell, B. G., Siegel, D. A., Carder, K. L., Garver, S. A., . . . McClain, C. (1998). Ocean color chlorophyll algorithms for SeaWiFS. *J. Geophys. Res. Oceans, 103*(C11), 24937-24953. doi:10.1029/98JC02160

Werdell, P. J., & Bailey, S. W. (2005). An improved in-situ bio-optical data set for ocean color algorithm development and satellite data product validation. *Remote Sensing of Environment, 98*(1), 122-140. doi:http://dx.doi.org/10.1016/j.rse.2005.07.001

Appendix B: Diffuse attenuation coefficient and uncertainty

NASA's standard algorithm for the deriving diffuse attenuation coefficient at 490 nm, K_{d},490 (m^{-1}), is based on blue-to-green reflectance ratios (Mueller, 2000). The algorithm was empirically developed using a high quality in situ dataset of coincident $K_d(490)$ and $R_{rs}(\lambda)$ data (Mueller, 2000; Werdell & Bailey, 2005) and is computed as follows:

$$K_{d,490} = 0.0166 + 10^{\chi} \quad \text{[B1]}$$

which has the derivative with respect to χ of:

$$\frac{\partial K_{d,490}}{\partial \chi} = \log(10)10^{\chi} \quad \text{[B2]}$$

where χ is a polynomial function. The order of the polynomial, $N=4$, and the coefficients b_{i} are sensor dependent. Specifically, χ is expressed as:

$$\chi = \sum_{i=0}^{4} b_i \left[LR\right]^i \quad \text{[B3]}$$

and has the derivative (assuming the coefficients, b_i, have no uncertainties):

$$\frac{\partial \chi}{\partial LR} = \sum_{i=3}^{4} i b_i \left[LR\right]^{i-1} \ . \quad \text{[b4]}$$

In this study, we consider the $K_{d,490}$ algorithm tuned for SeaWiFS such that the blue and green remote sensing reflectances, $R_{rs,b}$ and $R_{rs,g}$, that are centered on 490 nm and 555 nm, respectively. Also, the fourth order polynomial coefficients b_0, b_1, b_2, b_3, and b_4 are -0.8515, -1.8263, 1.8714, -2.4414, and -1.0690, respectively. Thus, the log-ratio, LR, term is:

$$LR = \log_{10}\left(\frac{R_{rs,490}}{R_{rs,555}}\right) \quad \text{[B5]}$$

The partial derivatives of Eq. B5 are:

$$\frac{\partial LR}{\partial R_{rs,490}} = \frac{1}{\log(10)} \frac{1}{R_{rs,555}} \ , \quad \text{[B6a]}$$

and

$$\frac{\partial LR}{\partial R_{rs,555}} = -\frac{1}{\log(10)} \frac{R_{rs,490}}{R_{rs,555}} \ . \quad \text{[B6b]}$$

The variance in $K_{d,490}$ can thus be estimated as:

$$u^2(K_{d,490}) = \left(\frac{\partial K_{d,490}}{R_{rs,490}}\right)^2 u^2(R_{rs,490}) + \left(\frac{\partial K_{d,490}}{R_{rs,555}}\right)^2 u^2(R_{rs,555}) + 2\frac{\partial K_{d,490}}{R_{rs,490}}\frac{\partial K_{d,490}}{R_{rs,555}} u(R_{rs,490}, R_{rs,555})$$

[B8]

where u($R_{rs,490}$) and u($R_{rs,555}$) are the variances of $R_{rs,490}$ and $R_{rs,555}$, respectively and $u(R_{rs,490}, R_{rs,555})$ is the error covariance of $R_{rs,490}$ and $R_{rs,555}$. The partial derivatives in Eq. B8 are computed as:

$$\frac{\partial K_{d,490}}{\partial R_{rs,490}} = \frac{\partial K_{d,490}}{\partial \chi}\frac{\partial \chi}{\partial LR}\frac{\partial LR}{\partial R_{rs,490}} \quad \text{[B9a]}$$

and

$$\frac{\partial K_{d,490}}{\partial R_{rs,555}} = \frac{\partial K_{d,490}}{\partial \chi}\frac{\partial \chi}{\partial LR}\frac{\partial LR}{\partial R_{rs,555}}. \quad \text{[B9b]}$$

References

Mueller, J. L. (2000). SeaWiFS algorithm for the diffuse attenuation coefficient, K(490), using water-leaving radiances at 490 and 555 nm. In S. B. Hooker & E. Firestone, R (Eds.), *NASA Technical Memorandum 2000-206829* (Vol. 11, pp. 51). Greenbelt, USA: NASA Goddard Space Flight Center.

Werdell, P. J., & Bailey, S. W. (2005). An improved in-situ bio-optical data set for ocean color algorithm development and satellite data product validation. *Remote Sensing of Environment, 98*(1), 122-140. doi:http://dx.doi.org/10.1016/j.rse.2005.07.001

Appendix C: Particulate organic carbon and uncertainty

NASA's Particulate Organic Carbon (*POC*) algorithm as defined by Stramski et al. (2008a) computes near-surface particular organic carbon concentration (mg m^{-3}) as follows:

$$POC = a_{poc} BR^{b_{poc}} \qquad [C1]$$

Where, a_{poc} and b_{poc} are constants with values of 203.2 and -1.034, respectively. The *BR* term is a blue-green reflectance ratio with the numerator and denominator being remote sensing reflectances centered on 443 and 555 nm, respectively.

$$BR = \frac{R_{rs,443}}{R_{rs,555}} \qquad [C2]$$

The derivative of Equation C1 with respect to *BR* is:

$$\frac{\partial POC}{\partial BR} = a_{poc} b_{poc} BR^{(b_{poc}-1)}, \qquad [C3]$$

and Equation C2 had the following partial derivatives:

$$\frac{\partial BR}{\partial R_{rs,443}} = \frac{1}{R_{rs,555}}, \qquad [C4]$$

and

$$\frac{\partial BR}{\partial R_{rs,555}} = -\frac{R_{rs,443}}{(R_{rs,555})^2}. \qquad [C5]$$

The variance of *POC* is estimated as:

$$u^2(POC) = \left(\frac{\partial POC}{\partial R_{rs,443}}\right)^2 u^2(R_{rs,443}) + \left(\frac{\partial POC}{\partial R_{rs,555}}\right)^2 u^2(R_{rs,555}) + 2\frac{\partial POC}{\partial R_{rs,443}}\frac{\partial POC}{\partial R_{rs,555}} u(R_{rs,443}, R_{rs,555})$$

[C5]

where $u^2(R_{rs,443})$ and $u^2(R_{rs,555})$ are variances of $R_{rs,443}$ and $R_{rs,555}$, respectively. $u(R_{rs,443}, R_{rs,555})$ is the error covariance of $R_{rs,443}$ and $R_{rs,555}$. The partial derivatives in Eq. C5 are computed as:

$$\frac{\partial POC}{\partial R_{rs,443}} = \frac{\partial POC}{\partial BR}\frac{\partial BR}{\partial R_{rs,443}}, \qquad [C6a]$$

and

$$\frac{\partial POC}{\partial R_{rs,555}} = \frac{\partial POC}{\partial BR}\frac{\partial BR}{\partial R_{rs,555}}. \qquad [C6b]$$

References

Stramski, D., Reynolds, R. A., Babin, M., Kaczmarek, S., Lewis, M. R., Rottgers, R., . . . Claustre, H. (2008a). Relationships between the surface concentration of particulate organic carbon and optical properties in the eastern South Pacific and eastern Atlantic Oceans. *Biogeosciences, 5*(1), 171-201. doi:10.5194/bg-5-171-2008

Appendix D: Normalized fluorescent line height and uncertainty

NASA's algorithm for normalized fluorescence line height, *nflh* (mW cm^{-2} µm^{-1} sr^{-1}), is a measurement of chlorophyll fluorescence emission under natural sunlight (Behrenfeld et al., 2009). The algorithm uses spectral values of normalized water leaving radiances, *nLw*. Values of *nflh* are calculated as the difference between the observed nLw_{678} and a linearly interpolated nLw_{678} from two adjacent bands (nLw_{667} and nLw_{748}). Currently, the algorithm is implemented for MODIS only as:

$$nflh = nLw_{678} - nLw_{667}\left(\frac{70}{81}\right) - nLw_{748}\left(\frac{11}{81}\right). \qquad \text{[D1]}$$

We note that nLw_i is related to $R_{rs,i}$ as follows:

$$nLw_i = F_{0,i} R_{rs,i} \;, \qquad \text{[D2]}$$

where, $F_{0,i}$ is the spectral extraterrestrial solar irradiance (Thuillier et al., 2003).

The variance in *nflh* is estimated as (assuming that F_0 has no uncertainties):

$$\begin{aligned} u^2(nflh) = &\left(\frac{\partial nflh}{\partial R_{rs,667}}\right)^2 u^2(R_{rs,667}) + \left(\frac{\partial nflh}{\partial R_{rs,678}}\right)^2 u^2(R_{rs,678}) + \left(\frac{\partial nflh}{\partial R_{rs,748}}\right)^2 u^2(R_{rs,748}) \ldots \\ &+ 2\frac{\partial nflh}{\partial R_{rs,667}}\frac{\partial nflh}{\partial R_{rs,678}} u(R_{rs,667}, R_{rs,678}) + 2\frac{\partial nflh}{\partial R_{rs,667}}\frac{\partial nflh}{\partial R_{rs,748}} u(R_{rs,667}, R_{rs,748}) \ldots \\ &+ 2\frac{\partial nflh}{\partial R_{rs,678}}\frac{\partial nflh}{\partial R_{rs,748}} u(R_{rs,678}, R_{rs,748}) \end{aligned}$$

[D3]

where $u(R_{rs,667})$, $u(R_{rs,678})$, and $u(R_{rs,748})$ are the uncertainties in $R_{rs,667}$, $R_{rs,678}$, and $R_{rs,748}$, respectively. The term $u(R_{rs,667}, R_{rs,678})$ is the error covariance of $R_{rs,667}$ and $R_{rs,678}$, $u(R_{rs,667}, R_{rs,748})$ is the error covariance of $R_{rs,667}$ and $R_{rs,748}$, and $(R_{rs,678}, R_{rs,748})$ is the error covariance of $R_{rs,667}$, and $R_{rs,748}$. The partial derivatives in Eq. D3 are:

$$\frac{\partial nflh}{\partial R_{rs,667}} = -\left(\frac{70}{81}\right) F_{0,667} \;, \qquad \text{[D4a]}$$

$$\frac{\partial nflh}{\partial R_{rs,678}} = F_{0,678} \;, \qquad \text{[D4b]}$$

and

$$\frac{\partial nflh}{\partial R_{rs,748}} = -\left(\frac{11}{81}\right) F_{0,748} \;. \qquad \text{[D4c]}$$

References

Behrenfeld, M. J., Westberry, T. K., Boss, E. S., O'Malley, R. T., Siegel, D. A., Wiggert, J. D., . . . Mahowald, N. (2009). Satellite-detected fluorescence reveals global physiology of ocean phytoplankton. *Biogeosciences, 6*(5), 779-794. doi:10.5194/bg-6-779-2009

Thuillier, G., Hersé, M., Labs, D., Foujols, T., Peetermans, W., Gillotay, D., . . . Mandel, H. (2003). The Solar Spectral Irradiance from 200 to 2400 nm as Measured by the SOLSPEC Spectrometer from the Atlas and Eureca Missions. *Solar Physics, 214*(1), 1-22. doi:10.1023/A:1024048429145

Appendix E: Inherent optical properties and uncertainty

The Generalized Inherent Optical Properties (GIOP) is a semianalytical algorithm used to derive standard IOP data products as distributed by NASA's OB.DAAC. Comprehensive discussion of the GIOP can be found elsewhere (Franz & Werdell, 2010; McKinna et al., 2016; Werdell et al., 2013), however, below we briefly overview the algorithm.

E.1 The forward model

At the core of the GIOP is a forward reflectance model that simulates the spectral sub-surface remote-sensing reflectance, $r_{rs,i}$, as a function of the water-column's inherent optical properties (IOPs). The default configuration of the GIOP uses the quasi-single scattering approximation of Gordon et al. (1988) to model the subsurface spectral remote-sensing reflectance, $r_{rs,i}^{\mathrm{mod}}$, as a function of IOPs:

$$r_{rs,i}^{\mathrm{mod}} = g_0 u_i + g_1 u_i^2, \quad \text{[E1]}$$

and

$$u_i = \frac{b_{b,i}}{a_i + b_{b,i}} \quad \text{[E2]}$$

where, a_i is the total spectral absorption coefficient, $b_{b,i}$ is the total spectral backscattering coefficient, and g_0 and g_1 are constants with default values of 0.0949 and 0.0794, respectively. The coefficient a_i can be expressed as the sum of absorbing constituent matter present:

$$a_i = a_{w,i} + x_\phi a_{\phi,i}^* + x_{dg} a_{dg,i}^* \quad \text{[E3]}$$

where, the $a_{w,i}$ is the spectral absorption coefficient of pure water. The two remaining spectral absorption coefficient terms on the right-hand side of Equation E3 are expressed as a product of a normalized spectral absorption coefficient ($a*$) and its magnitude (x). The subscripts ϕ, and dg denote the constituents phytoplankton and colored dissolved and detrital matter, respectively. Similarly, $b_{b,i}$ can be expressed as:

$$b_{b,i} = b_{bw,i} + x_p b_{bp,i}^* \quad \text{[E4]}$$

where the subcomponents of water and particulate matter are denoted by the subscripts w and p. Because pure water IOPs and the spectral shapes of other constituent matter can be parameterized at runtime, $r_{rs,i}^{\mathrm{mod}}$ becomes a function of three free variables:

$$r_{rs,i}^{\mathrm{mod}} = f(x_\phi, x_{dg}, x_p). \quad \text{[E5]}$$

A mathematical solution method (default: non-linear least squares optimization) is then employed to find the optimal set of x_φ, x_{dg}, and x_p such that $r_{rs,i}^{\mathrm{mod}}$ best matches the sensor-observed sub-surface remote-sensing reflectance, $r_{rs,i}^{\mathrm{mod}}$. A "best match" is achieved once some distance metric (e.g. chi-squared) falls below a predefined threshold. We note that $r_{rs,i}^{\mathrm{mod}}$ is computed from above-water remote sensing reflectance, $R_{rs,i}$ according to Lee et al. (Lee et al., 2002):

$$r_{rs,i}^{obs} = \frac{R_{rs,i}}{0.52 + 1.7 R_{rs,i}} . \quad \text{[E6]}$$

Importantly, the GIOP's structure can be varied at runtime to assign the forward reflectance model, the normalized shapes of the IOP subcomponents ($a_{\phi,i}^{*}$, $a_{dg,i}^{*}$ and $b_{bp,i}^{*}$), and the mathematical solution method. We note that the spectral shape coefficients are normalized at 443 nm.

E.2 Bio-optical models

In the GIOP, the normalized shape components $a_{\phi,i}^{*}$, $a_{dg,i}^{*}$ and $b_{bp,i}^{*}$ are parameterized on a per-pixel basis using bio-optical models. Below we briefly describe the bio-optical models used in the default configuration of the GIOP.

The spectral shape $a_{\phi,i}^{*}$ is modeled per-pixel using the methodology of Bricaud et al. (1998). Specifically, $a_{\phi,i}^{*}$ is a function of *Chl* as derived in Appendix A and the spectral vectors A_i and B_i:

$$a_{\phi,i}^{*} = \frac{0.055}{\tau} A_i Chl^{B_i - 1}, \quad \text{[E7]}$$

where, the scaling coefficient is:

$$\tau = A_{440} Chl^{B_{440} - 1}. \quad \text{[E8]}$$

The subscript 440 denotes that the scaling coefficient is computed at or near 440 nm. The resulting $a_{\phi,i}^{*}$ is chlorophyll-specific, hence the scaling factor x_{ϕ} has the physical value of chlorophyll concentration (Chl_{giop}). The spectral shape $a_{dg,i}^{*}$ is modeled using an exponential function of the form:

$$a_{dg,i}^{*} = \exp\left\{-S_{dg}(i - 440)\right\} \quad \text{[E9\}}$$

where, S_{dg} is treated as a constant with a default value of 0.0183 sr^{-1} (Werdell et al., 2013). The normalized spectral shape has a value of 1.0 at or near 440 nm. Accordingly, the scaling factor x_{dg} is equivalent to $a_{dg,440}$.

The normalized particulate backscattering coefficient, $b_{bp,i}^{*}$, is modeled per-pixel using a power law:

$$b_{bp,i}^{*} = \left(\frac{440}{i}\right)^{\gamma} . \quad \text{[E10]}$$

The normalized spectral shape has a value of 1.0 at or near 440 nm. The power law exponent, γ, is calculated following Lee et al. (2002):

[E11]

where

$$v = -0.9\left(\frac{r_{rs,440}}{r_{rs,550}}\right), \quad \text{[E12]}$$

and $r_{rs,550}$ is centered on or near 550 nm.

E.3 Inverse solution method

The GIOP has a number of built-in inverse solution (spectral matching) methods that an end-user can select at runtime, these including: Levenberg-Marquardt (LM) optimization, Nelder-Mead (amoeba) optimization, and linear matrix inversion (LMI). In this study, we have chosen to use a LM solution method as is it is the current default in NASA's implementation of GIOP. The cost function is a Chi-squared sum of squares metric. When computing the Chi-squared metric, the following $R_{rs,i}$ bands were included: 412, 425, 443, 460, 475, 490, 510, 532, 555, 583, 617, 640, 655, 665 nm.

E.4 Uncertainty propagation

For non-linear least squares, the variance-covariance matrix of derived best-fit parameters, $\mathbf{V_x}$, can be estimated using the Jacobian matrix, **J**, and the variance-covariance matrix of model inputs, $\mathbf{V_{rrs}}$ as:

$$\mathbf{V}_x = \mathbf{J}^{-1}\mathbf{V}_{\mathbf{r_{rs}}}(\mathbf{J}^{\mathrm{T}})^{-1} . \quad \text{[E13]}$$

For the GIOP, the uncertainties of x_ϕ, x_{dg}, and x_p can thus be estimated as the square root of the diagonal elements of $\mathbf{V_x}$. The matrix $\mathbf{V}_{\mathbf{r_{rs}}}$ is the variance-covariance matrix of r_{rs}. The matrix **J** is computed as:

$$\mathbf{J} = \left[\frac{\partial r_{rs,i}}{\partial x_\phi} \quad \frac{\partial r_{rs,i}}{\partial x_{dg}} \quad \frac{\partial r_{rs,i}}{\partial x_p} \right] \quad \text{[E14]}$$

where the ith wavelength element of each column can be expressed as:

$$\frac{\partial r_{rs,i}}{\partial x_\phi} = \frac{-[g_0 + 2g_1u_i][b_{b,i}a^*_{\phi,i}]}{[b_{b,i} + a_i]^2} , \quad \text{[E15a]}$$

$$\frac{\partial r_{rs,i}}{\partial x_{dg}} = \frac{-[g_0 + 2g_1u_i][b_{b,i}a^*_{dg,i}]}{[b_{b,i} + a_i]^2} , \quad \text{[E15b]}$$

and

$$\frac{\partial r_{rs,i}}{\partial x_p} = \frac{[g_0 + 2g_1u_i][a_ib^*_{bp,i}]}{[b_{b,i} + a_i]^2} . \quad \text{[E15c]}$$

The diagonal elements of $\mathbf{V}_{\mathbf{r_{rs}}}$ are equal to the square of uncertainties in sensor-observed sub-surface remote sensing reflectances, $u^2(r_{rs,i})$, and the off-diagonal elements of $\mathbf{V}_{\mathbf{r_{rs}}}$, $u(r_{rs,i}, r_{rs,j})$, are the covariances between $r_{rs,i}$ and $r_{rs,j}$. The elements $u^2(r_{rs,i})$ are computed as:

$$u^2(r_{rs,i}) = \left(\frac{\partial r_{rs,i}}{\partial R_{rs,i}}\right)^2 u^2(R_{rs,i}), \quad \text{[E16]}$$

and the off-diagonal can be computed as:

$$u(r_{rs,i}, r_{rs,j}) = \frac{\partial r_{rs,i}}{\partial R_{rs,i}} \frac{\partial r_{rs,i}}{\partial R_{rs,j}} u(R_{rs,i}, R_{rs,j}) \quad \text{[E17]}$$

where, $u(R_{rs,i}, R_{rs,j})$ is the covariance (if known) of above-water remote sensing reflectances $R_{rs,i}$ and $R_{rs,j}$. The partial derivative term in E16 is:

$$\frac{\partial r_{rs,i}}{\partial R_{rs,i}} = \frac{0.52}{\left[0.52 + 1.7 R_{rs,i}\right]^2} \ . \quad \text{[E18]}$$

We note that for the approximation of $u(x_\phi)$, $u(x_{dg})$ and $u(x_p)$, computed from Eq. E13, the spectral shapes coefficients $a^*_{\phi,i}$, $a^*_{dg,i}$, and $b^*_{bp,i}$ are treated as uncertainty free. In practice, however, we note that $a^*_{\phi,i}$ and $b^*_{bp,i}$ have inherent uncertainties due to their dependence of *Chl* and $R_{rs,i}$, respectively. We remind the reader that in this study we have focused on propagation of data (radiometric) uncertainties and not on the impact of model (spectral shape) uncertainties. We have nonetheless still included relevant FOFM uncertainty formulations for spectral shape models in the following section.

E.5 Uncertainty in spectral shapes

For $b^*_{bp,i}$, variance of the spectral shape, $u^2(b^*_{bp,i})$, is driven by variance in the power law exponent, $u^2(\gamma)$, which can be computed as:

$$u^2(\gamma) = \left(\frac{\partial \gamma}{\partial r_{rs,440}}\right)^2 u^2(r_{rs,440}) + \left(\frac{\partial \gamma}{\partial r_{rs,550}}\right)^2 u^2(r_{rs,550}) + 2\frac{\partial \gamma}{\partial r_{rs,440}} \frac{\partial \gamma}{\partial r_{rs,550}} u(r_{rs,440}, r_{rs,550}) \, .$$

[E19]

The partial derivatives in Eq. E19 have the following form:

$$\frac{\partial \gamma}{\partial r_{rs,440}} = \frac{\partial \gamma}{\partial \nu} \frac{\partial \nu}{\partial r_{rs,440}} \ , \quad \text{[E20a]}$$

$$\frac{\partial \gamma}{\partial r_{rs,550}} = \frac{\partial \gamma}{\partial \nu} \frac{\partial \nu}{\partial r_{rs,550}} , \quad \text{[E20b]}$$

where,

$$\frac{\partial \gamma}{\partial v} = -2.4e^{v} \ , \qquad \text{[E21]}$$

$$\frac{\partial v}{\partial r_{rs,440}} = -\frac{0.9}{r_{rs,440}} \ , \qquad \text{[E22]}$$

and

$$\frac{\partial v}{\partial r_{rs,550}} = 0.9\frac{r_{rs,440}}{(r_{rs,555})^2} . \qquad \text{[E23]}$$

Finally, $u(b^*_{bp,i})$ is computed as:

$$u(b^*_{bp,i}) = u(\gamma)\log\left(\frac{440}{\lambda}\right)\left(\frac{440}{\lambda}\right)^{\gamma} \qquad \text{[E24]}$$

and spectral uncertainty in the derived backscattering coefficient, $u(b_{bp,i})$, is:

$$u^2(b_{bp,i}) = \left(x_p u(b^*_{bp,i})\right)^2 + \left(b^*_{bp,i} u(x_p)\right)^2 + 2x_p b^*_{bp,i} u(x_p, b^*_{bp,i}) \qquad \text{[E25]}$$

where the term $u(x_p, b^*_{bp,i})$ is the covariance between x_p and $b^*_{bp,i}$. When estimating $u(b_{bp,443})$ the first and third term in the right-hand side of E25 reduces to zero as $b^*_{bp,443}$ has a constant value of 1.0 and thus no variance, hence $u(x_p, b^*_{bp,443})$ is zero. We have not estimated/parameterized $u(x_p, b^*_{bp,i})$ in this study.

For $a^*_{\phi,i}$, uncertainty of the spectral shape, $u^2(a^*_{\phi,i})$, is driven by $u^2(Chl)$ and is computed as:

$$u^2(a^*_{\phi,i}) = \left(\frac{\partial a^*_{\phi}(\lambda)}{\partial Chl}\right)^2 u^2(Chl) \qquad \text{[E26]}$$

where,

$$\frac{\partial a^*_{\phi,i}}{\partial Chl} = \frac{0.055}{\tau}\frac{\partial A_i Chl^{B_i-1}}{\partial Chl} + A_i Chl^{B_i-1}\frac{\partial}{\partial Chl}\left(\frac{0.055}{\tau}\right) . \qquad \text{[E27]}$$

The term in Eq. E27 are expressed as:

$$\frac{\partial A_i Chl^{B_i-1}}{\partial Chl} = \left[E_i - 1\right] A_i Chl^{B_i-2} \qquad \text{[E28]}$$

and

$$\frac{\partial}{\partial Chl}\frac{0.055}{\tau}=\frac{\partial}{\partial Chl}\frac{0.055}{A_{440}Chl^{B_{440}-1}}=\frac{0.055\left[-B_{440}+1\right]}{A_{440}Chl^{B_{440}}} \;. \qquad \text{[E29]}$$

Spectral variance in the derived phytoplankton absorption coefficient, $u^2(a_{\phi,i})$, is:

$$u^2(a_{\phi,i})=\left(x_\phi u(a^*_{\phi,i})\right)^2+\left(a^*_{\phi,i}u(x_\phi)\right)^2+2x_\phi a^*_{\phi,i}u(x_\phi,a^*_{\phi,i}) \;, \qquad \text{[E30]}$$

where the term $u(x_\phi,a^*_{\phi,i})$is the covariance between x_ϕ and $a^*_{\phi,i}$. When estimating $u(a_{\phi,443})$ the first and third terms in the right-hand side of E30 reduces to zero as $a^*_{\phi,443}$ has a constant value of 0.055 m^2 mg^{-1} and thus no variance, hence $u(x_\phi,a^*_{\phi,i})$ is zero. We have not estimated/parameterized $u(x_\phi,a^*_{\phi,i})$ in this study. We also note the spectral coefficients *A, B,* and the scaling constant of 0.055 each would have asscociated uncertainties, however, quanitifying these model coefficient ucertainties was beyond the scope of this work.

The spectral variance in the absorption coefficient of colored dissolved and detrital matter, $u^2(a_{dg,i})$, can be estimated as:

$$u^2(a_{dg,i})=\left(a^*_{dg,i}u(x_{dg})\right)^2+\left(x_{dg}u(a^*_{dg,i})\right)^2+2x_{dg}a^*_{dg,i}u(x_{dg},a^*_{dg,i}) \;. \qquad \text{[E31]}$$

We note that in the default parametrization of the GIOP, S_{dg} is treated as a constant, thus there is no variance in $a^*_{dg,i}$, and the first and last terms therefore reduce to zero.

References

Bricaud, A., Morel, A., Babin, M., Allali, K., & Claustre, H. (1998). Variations of light absorption by suspended particles with the chlorophyll a concentration in oceanic (Case 1) waters : analysis and implications for bio-optical models. *J. Geophys. Res. Oceans, 103*, 31,033 - 031,044.

Franz, B. A., & Werdell, P. J. (2010). A Generalized Framework for Modeling of Inherent Optical Propertiesin Ocean Remote Sensing Applications. *Proc. Ocean Optics XX, Anchorage, Alaska, USA, 27 September - 1 October.*

Gordon, H. R., Brown, O. B., Evans, R. H., Brown, J. W., Smith, R. C., Baker, K. S., & Clark, D. K. (1988). A semianalytic radiance model of ocean color. *J. Geophys. Res. Atmos., 93*(D9), 10909-10924. doi:10.1029/JD093iD09p10909

Lee, Z., Carder, K. L., & Arnone, R. A. (2002). Deriving inherent optical properties from water color: a multiband quasi-analytical algorithm for optically deep waters. *Applied Optics, 41*(27), 5755-5772. doi:10.1364/AO.41.005755

McKinna, L. I. W., Werdell, P. J., & Proctor, C. W. (2016). Implementation of an analytical Raman scattering correction for satellite ocean-color processing. *Optics Express, 24*(14), A1123-A1137. doi:10.1364/OE.24.0A1123

Werdell, P. J., Franz, B. A., Bailey, S. W., Feldman, G. C., Boss, E., Brando, V. E., . . . Lee, Z. (2013). Generalized ocean color inversion model for retrieving marine inherent optical properties. *Applied Optics, 52*(10), 2019-2037.

Appendix F: Approximating correlated uncertainties due to sensor noise

For this study, we have statistically estimated the spectral covariance matrix for SeaWiFS remote sensing reflectances $\mathbf{V}_{Rrs}$ due to radiometric uncertainty in L_t. The objective was to appraise the impact of spectral covariance terms in the analytical FOFM uncertainty framework, not to exactly quantify the covariances for any given sensor. We accept that it does not encompass a wide variety of viewing geometries, scan angles, water types, and aerosol conditions. In addition, we have not considered the uncertainties in ancillary datasets such ozone concentration, and wind speed. This methodology followed two steps: (i) statistically derive $\mathbf{V}_{Rrs}$ from a number of SeaWiFS images captured over the South Pacific Gyre, and (ii) numerically estimate the Jacobian matrix, $\mathbf{J}_{\mathbf{Lt}}$, describing how small changes in L_t affect derived R_{rs}. The SPG was selected as atmospheric and oceanic gradients in this region can be considered quasi-homogenous at local horizontal scales, thus local variability in $L_{t,i}$ can, to a first order, be attributed to sensor noise. We have used SeaWiFS MLAC data distributed by NASA's Ocean Biology Distributed Active Archive and Center (OB.DAAC) and processed these using NASA's l2gen code which is distributed as part of the SeaDAS data processing and visualization software package (https://seadas.gsfc.nasa.gov/). Following JCGM (2008) we have applied correction factors, including vicarious calibration and temporal degradation gains, in an attempt to reduce systematic effects.

First, we followed a similar approach to Lamquin et al. (2013) to statistically estimate the covariance matrix of top-of-atmosphere radiances, $\mathbf{V}_{Lt}$. A selection of 1,928 SeaWiFS MLAC level-1 files spanning the years 1999 – 2010 were processed using l2gen. Each L1 MLAC file was encompassed, in part or whole, a 1° x 1° region centered on 26°S, 122°E in the SPG. The derived level-2 (L2) data products were: $L_{t,i}$, $R_{rs,i}$ and *Chl*. The quality of each level-2 file was then assessed by examining the proportion of a file flagged as: CLDICE, HIGLINT, or PRODFAIL (probable cloud or ice contamination, sunglint: reflectance exceeds threshold, and failure in any product, respectively). This reduced the number of L2 files to 188. Next for each of the 188 L2 SPG extract files were manually inspected in SeaDAS v7.5, and depending on their size, one-to-three 5x5 pixel subsets were extracted where *Chl* and $R_{rs,510}$ appeared to be quasi-homogenous, resulting in a total of 212 L2 5x5-pixel subset files. Finally, the covariance matrix of L_t was computed for each 5x5 pixel subset from which a median average $\mathbf{V}_{Lt}$ was generated. Using $\mathbf{V}_{Lt}$ the correlation matrix $\mathbf{R}_{Lt}$ was then computed. Figure F1 shows estimated $\mathbf{V}_{Lt}$ and $\mathbf{R}_{Lt}$.

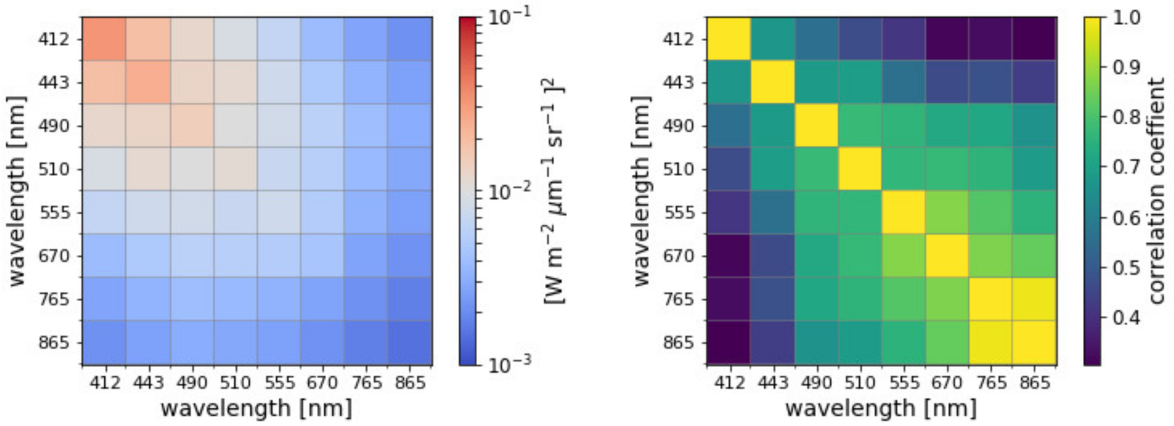

Figure F1: Left-hand side: estimated variance-covariance matrix of SeaWiFS top-of-atmosphere radiances, V_{Lt}. Right-hand side: estimated correlation matrix of SeaWiFS top-of-atmosphere radiances, R_{Lt}.

In this study, we approximated the per band top-of-atmosphere radiance uncertainty $u(L_{t,i})$ as 0.5% of total $L_{t,i}$:

$$u_n(L_{t,i}) = 0.005 \times L_{t,i}. \quad \text{[F2]}$$

Next, we estimate a scaled top-of-atmosphere covariance matrix, $\mathbf{V}'_{Lt}$, on a pixel-by-pixel basis which has diagonal elements of $u^2(L_{t,i})$ and can be computed as:

$$\mathbf{V}'_{Lt} = \mathbf{S}\mathbf{R}_{Lt}\mathbf{S}, \quad \text{[F3]}$$

where the matrix **S** is a diagonal matrix with elements equal to $1/u_n(L_{t,i})$.

A comprehensive method to propagate radiometric uncertainties through NASA's standard atmospheric correction algorithm was beyond the scope of this study. We instead used a numerical approach to approximate the covariance matrix of sensor-derived remote sensing reflectances, $\mathbf{V}_{Rrs}$. We first numerically estimated the Jacobian matrix, $\mathbf{J}_{Lt}$, or partial derivatives of $R_{rs,i}$ (412 – 670 nm) with respect to $L_{t,i}$ (412 – 865 nm)

$$\mathbf{J}_{Lt} = \begin{bmatrix} \frac{\partial R_{rs,412}}{\partial L_{t,412}}, & \frac{\partial R_{rs,412}}{\partial L_{t,443}}, & \cdots & \frac{\partial R_{rs,412}}{\partial L_{t,865}} \\ \frac{\partial R_{rs,443}}{\partial L_{t,412}}, & \frac{\partial R_{rs,443}}{\partial L_{t,443}}, & \cdots & \frac{\partial R_{rs443}}{\partial L_{t,865}} \\ \vdots & \vdots & & \vdots \\ \frac{\partial R_{rs,670}}{\partial L_{t412}}, & \frac{\partial R_{rs,670}}{\partial L_{t,443}}, & \cdots & \frac{\partial R_{rs,670}}{\partial L_{t,865}} \end{bmatrix}. \quad \text{[F4]}$$

To generate $\mathbf{J}_{Lt}$, for the SeaWiFS MLAC image captured on 14 March 2007 and centered on the SPG, a subset of the image was extracted (25°S—27°S; 121.3°W -123.3°) with mean solar zenith angle (σ_{solz}) of 24.5° (1-σ_{solz}=0.69°) and mean sensor zenith angle (σ_{senz}) of 23.9° (1-σ_{senz}=1.9°). The subset was then processed using l2gen to derive $R_{rs,i}$ Next, the scene was reprocessed, however, this time $L_{t,412}$ was perturbed by a 0.1% of the average scene-wide value of $L_{t,412}$. The perturbation process was performed eight times, once for each spectral band. Finally, the partial derivatives in $\mathbf{J}_{Lt}$ were approximated numerically using a finite difference method, for example:

$$\frac{\partial R_{rs,443}}{\partial L_{t,443}} \approx \frac{R'_{rs,443} - R_{rs,443}}{\Delta L_{t,443}} \quad \text{[F5]}$$

where, $R'_{rs,443}$ is the derived remote sensing reflectance at 443 derived when $L_{t,443}$ was perturbed by a small value $\Delta L_{t,443}$. Finally, $\mathbf{V}_{Rrs}$ can be estimated as:

$$\mathbf{V}_{Rrs} = \mathbf{J}_{Lt}\mathbf{V}'_{Lt}\mathbf{J}^{T}_{Lt}. \quad \text{[F6]}$$

An example $\mathbf{V}_{Rrs}$ matrix and example of $u(R_{r,is})$ spectra are shown in Figure F2. These data were derived for the test SeaWiFS MLAC scene captured on 14 March 2007 over the SPG. All elements of $\mathbf{V}_{Rrs}$ are positive. We note that the estimated relative uncertainties (Figure F2) are of similar in shape and magnitude to those reported by Hu et al. (2013) for low-*Chl* waters.

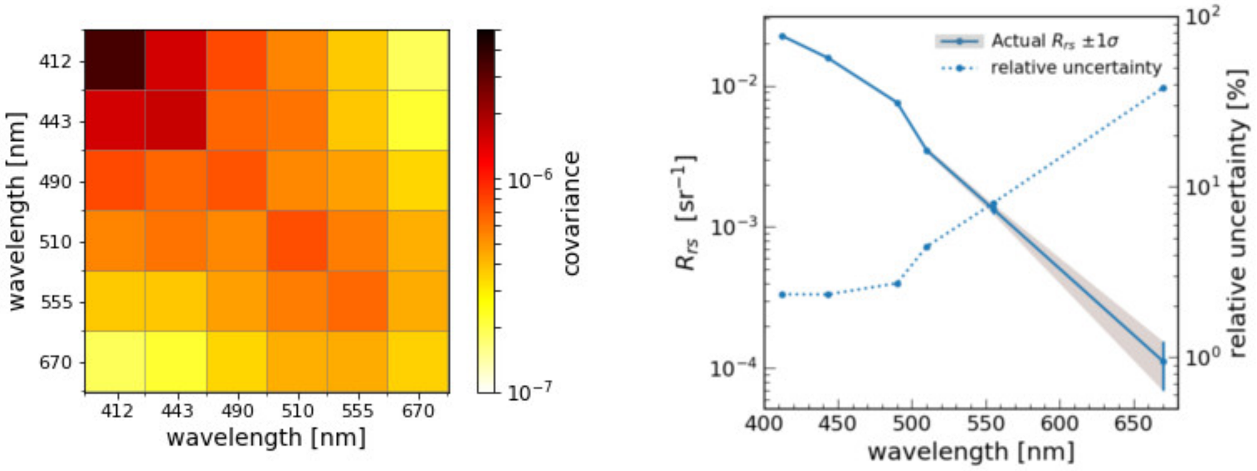

Figure F2: Left-hand side: estimated variance-covariance matrix of SeaWiFS remote sensing reflectances, V_{Rrs}, for a scene of the central SPG captured on 14 March 2007. Right-hand side: Solid blue line is depicting the average R_{rs} spectrum (log-scaled) for the SPG image with error bars that correspond to $u(R_{rs,i})$. Dashed line depicts relative uncertainty in R_{rs}.

References

Hu, C., Feng, L., & Lee, Z. (2013). Uncertainties of SeaWiFS and MODIS remote sensing reflectance: Implications from clear water measurements. *Remote Sensing of Environment, 133*, 168-182. doi:https://doi.org/10.1016/j.rse.2013.02.012

JCGM. (2008). *Evaluation of measurement data - Guide to the expression of uncertainty in measurement*. Retrieved from JCGM 100:2008:

Lamquin, N., Mangin, A., Mazeran, C., Bourg, B., Bruniquel, V., & D'Andon, O. F. (2013). *OLCI L2 pixel-by-pixel uncertainty propagation in OLCI clean water branch*: ESA ATBD ref. S3-L2-SD-01-C01-ACR-TN.
